# Active Microbial Airborne Dispersal and Biomorphs as Confounding Factors for Life Detection in the Cell-Degrading Brines of the Polyextreme Dallol Geothermal Field

**DOI:** 10.1128/mbio.00307-22

**Published:** 2022-04-06

**Authors:** Jodie Belilla, Miguel Iniesto, David Moreira, Karim Benzerara, Emmanuelle Gérard, José M. López-García, Electra Kotopoulou, Purificación López-García

**Affiliations:** a Ecologie Systématique Evolution, CNRS, Université Paris-Saclay, AgroParisTech, Orsay, France; b Institut de Minéralogie, de Physique des Matériaux et de Cosmochimie, CNRS, Sorbonne Université, Muséum National d’Histoire Naturelle, Paris, France; c Institut de Physique du Globe de Paris, CNRS, Université Paris Diderot, Paris, France; d Instituto Geológico y Minero de España, CSIC, Palma de Mallorca, Spain; University of Massachusetts Amherst

**Keywords:** extremophile, hyperacidity, hypersaline, hydrothermal, chaotropic, dispersal, archaea, life limits, biosignature, biomorph, contaminant

## Abstract

Determining the precise limits of life in polyextreme environments is challenging. Studies along gradients of polyextreme conditions in the Dallol proto-volcano area (Danakil salt desert, Ethiopia) showed the occurrence of archaea-dominated communities (up to 99%) in several hypersaline systems but strongly suggested that life did not thrive in the hyperacidic (pH ∼0), hypersaline (∼35% [wt/vol],) and sometimes hot (up to 108°C) ponds of the Dallol dome. However, it was recently claimed that archaea flourish in these brines based on the detection of one *Nanohaloarchaeotas* 16S rRNA gene and fluorescent *in situ* hybridization (FISH) experiments with archaea-specific probes. Here, we characterized the diversity of microorganisms in aerosols over Dallol, and we show that, in addition to typical bacteria from soil/dust, they transport halophilic archaea likely originating from neighboring hypersaline ecosystems. We also show that cells and DNA from cultures and natural local halophilic communities are rapidly destroyed upon contact with Dallol brine. Furthermore, we confirm the widespread occurrence of mineral particles, including silica-based biomorphs, in Dallol brines. FISH experiments using appropriate controls show that DNA fluorescent probes and dyes unspecifically bind to mineral precipitates in Dallol brines; cellular morphologies were unambiguously observed only in nearby hypersaline ecosystems. Our results show that airborne cell dispersal and unspecific binding of fluorescent probes are confounding factors likely affecting previous inferences of archaea thriving in Dallol. They highlight the need for controls and the consideration of alternative abiotic explanations before safely drawing conclusions about the presence of life in polyextreme terrestrial or extraterrestrial systems.

## INTRODUCTION

Determining the exact physicochemical limits of life on Earth is a difficult endeavor. Microbial life has adapted to a variety of extreme conditions (1), as exemplified by isolated microbial species growing at high temperatures (∼120°C) (2), at very low pH (∼0) (3), and under salt-saturating concentrations inducing low water activity in either NaCl-dominated (saturation at ∼5 M) or some MgCl_2_-enriched (4, 5) brines. However, in natural ecosystems at the boundary of life-permissive conditions, biomass is arguably very low, such that identifying autochthonous microbes and their biosignatures encounters difficulties comparable to those involved in deciphering unambiguous life traces in the oldest fossil record (6) or in a broader planetary exploration context (7). One major challenge is to establish rigorous criteria to differentiate bona fide cells (or microfossils) from biomorphs, i.e., abiotic structures that resemble them. Over the years, it has become clear that several purely geochemical processes can lead to the formation of biomorphic structures based on silica (8), sulfur (9), or carbonates (10). In particular hydrothermal and/or diagenetic contexts, these structures may even adsorb abiotically synthesized organics having isotopic compositions similar to those of biogenic compounds (11), further complicating disambiguation (6, 9).

Another important confounding factor is contamination. For example, in the earliest fossil record, foreign lipid biomarkers derived from drilling or migrating fluids mask potential fossil lipids syngenetic with the rock (12, 13). In modern low-biomass ecosystems, contamination with allochthonous cells muddles microbial diversity studies (14–17) and is a source of stochastic variation (18). Contamination may be introduced during sampling and laboratory manipulation (14, 19), even in clean environments such as spacecraft assembly facilities (20, 21), and/or derive from molecular biology kits (22, 23). At the same time, high-throughput sequencing methods have largely increased sensitivity for microbial diversity studies and, hence, have a greater potential to reveal contaminant sequences (24). Therefore, implementing controls when studying low-biomass ecosystems is essential, including blanks and methods to identify and, when possible, remove contaminants (14, 25, 26).

Contamination can also derive from naturally dispersing microbes in aerosols. The atmosphere ensures the connectivity of many ecosystems on Earth by facilitating dispersal even at very long distances (27–29). Intrinsic microbial properties, including size, shape, resistance to desiccation and UV radiation, and even mechanisms to cope with long-term starvation (30), influence dispersal, but so do local meteorological conditions. Accordingly, airborne microbial assemblages change temporally (31). Although most airborne microbes correspond to small coccoid microorganisms (<20-μm cell diameter), cells up to a few hundred micrometers can be transported as well (32). For instance, aeolian diatom dispersal over Antarctica is well documented (33). Desert dust and sandstorms are particularly well suited for dispersal and natural contamination (34, 35). For example, dust storms in Africa favor dispersal to Europe across the Mediterranean (36, 37); likewise, high loads of dust and airborne microbes have been observed over the Red Sea (38).

Airborne microbial dispersal and abiotic biomorph formation thus constitute challenging confounding factors for the precise delimitation of life limits in natural extreme settings at the surface of the planet. A remarkable example is offered by the geothermal field of Dallol and surrounding extreme environments in the salt desert of the North Danakil Depression, Ethiopia. The depression (∼120 m below sea level) extends over 200 km along the Afar rift at the junction between three main tectonic plates. It is covered by an ∼2-km-thick salt layer resulting from the evaporation of an ancient Red Sea arm separated from the main basin by volcanic deposits (39, 40). The Danakil depression has a hot desert climate (41) and is subject to strong, dust-bearing, hot dry winds locally named *hahaita-harrur* (fire wind) (42) and *gara* (fiery wind) (43). Dallol is a proto-volcanic salt dome located ∼30 km north of the hypersaline Lake Assale (As’ale or Karum), which borders the north end of the volcanic Erta Ale range. Dallol exhibits high continuous degassing and frequent hydrothermal activity depending on the interaction of infiltrating rainfall from the surrounding mountains with an underlying magmatic chamber (44, 45). Upwelling fluids enriched in magma-derived elements dissolve and incorporate diverse organics and salts as they traverse the overlying ancient marine sediments and the salt crust. This generates diverse hydrothermal manifestations on and around the dome of Dallol. This also influences the local hydrochemistry, leading to unique polyextreme conditions that, depending on the zone, combine hypersalinity (20 to >50% [wt/vol]), low pH (≤0 to 6.0), and high temperature (25 to 108°C) (39, 45–51).

Previous studies have unequivocally shown the presence of diverse microbial communities largely dominated by archaea in the hydrothermally influenced Lake Assale, the salt plain at the Dallol dome base, a salt-karst cave reservoir on the Dallol western canyons’ flank, and a series of small lakes located at the base of the Round Mountain (Western Canyon Lakes). These NaCl-dominated ecosystems, with ∼35% salinity, moderate temperature (∼30°C), and moderate pH (∼4 to 6), were largely enriched (85 to 99%) in highly diverse archaea, notably *Halobacteriota* and *Nanohaloarchaeota* (48, 51). In contrast, various lines of evidence on dozens of samples (cultures, 16S/18S rRNA gene metabarcoding, fluorescence-activated cell sorting, and optical and electron microscopy) failed to unambiguously identify microbial life thriving in two other types of brines in the Dallol area. One encompassed the Black and Yellow (Gaet’ale) lakes, located southwest and southeast of the dome, respectively, with lower pH (∼1.8 to 3) and higher temperature (40 to 70°C) and Mg^2+^-Ca^2+^-enriched salts (>50%). Water activity (a_w_, 0.261 to 0.467) and chaotropicity (198 to 320 kJ/kg) in these systems (48) were clearly incompatible with known limits for life (0.585 and 87.3 kJ/kg, respectively) (52–54). Water activity is an indicator of the availability of water molecules to hydrate and solubilize other molecules, whereas chaotropicity quantifies the disordering effects that some compounds, notably some salts, can have on macromolecules such as lipids or proteins (55).

The other seemingly lifeless environment corresponded to the highly acidic (pH ≤ 0), hypersaline (37 to 42%), and sometimes hot (∼30 to 108°C) Dallol dome colorful brines, enriched in Na^+^, Fe^2+/3+^, and Cl^−^. These displayed borderline a_w_ values for life (0.580 to 0.748) but permissive chaotropicity (−31.2 to +19.3 kJ/kg), although ferric chlorides, together with Mg and Ca chlorides, are highly chaotropic (56). The combination of these parameters with the possible presence of harmful chemical compounds appeared to prevent life from thriving under these conditions (48). However, another 16S rRNA gene amplicon study on two samples from the Dallol dome identified one operational taxonomic unit (OTU) affiliated with *Nanohaloarchaeota* on a salt chimney wall fragment. Although amplicons could not be produced from a neighboring pond, fluorescent *in situ* hybridization (FISH) seemingly indicated the specific presence of those archaea (57). In contrast, in our own 16S rRNA gene PCR amplification studies (48, 51), samples from the Black and Yellow lakes and Dallol failed to yield amplicons. Nonetheless, in several cases, after nested PCR reamplification, amplicons were produced from brines but only when first-PCR-negative controls yielded amplicons as well. Upon sequencing, the vast majority of reads were bacterial (95 to 100%), many typically associated with dust, humans, or molecular biology kits. Only a few sequences were archaeal and belonged to OTUs also found in Dallol-surrounding life-containing environments. Accordingly, we concluded that those archaeal OTUs were contaminants that had dispersed from nearby, less extreme hypersaline ecosystems (48).

In this study, we aimed at testing whether airborne microbial dispersal and mineral biomorphs, which are observed in many Danakil brines (48, 51), are confounding factors leading to the potentially mistaken detection of life in the Dallol ponds. We show not only that aerosols over the dome bear widely diverse cells but also that cells are rapidly destroyed by Dallol brines upon arrival. We also show that DNA fluorescent probes and dyes bind nonspecifically to cells and mineral precipitates under previously described FISH conditions (57). Our study highlights the need to include appropriate controls and consider alternative abiotic explanations before safely drawing conclusions about the presence of life in extreme terrestrial or extraterrestrial systems.

## RESULTS AND DISCUSSION

### Dallol aerosols transport widely diverse bacteria, eukaryotes, and some archaea.

**FIG 1:**
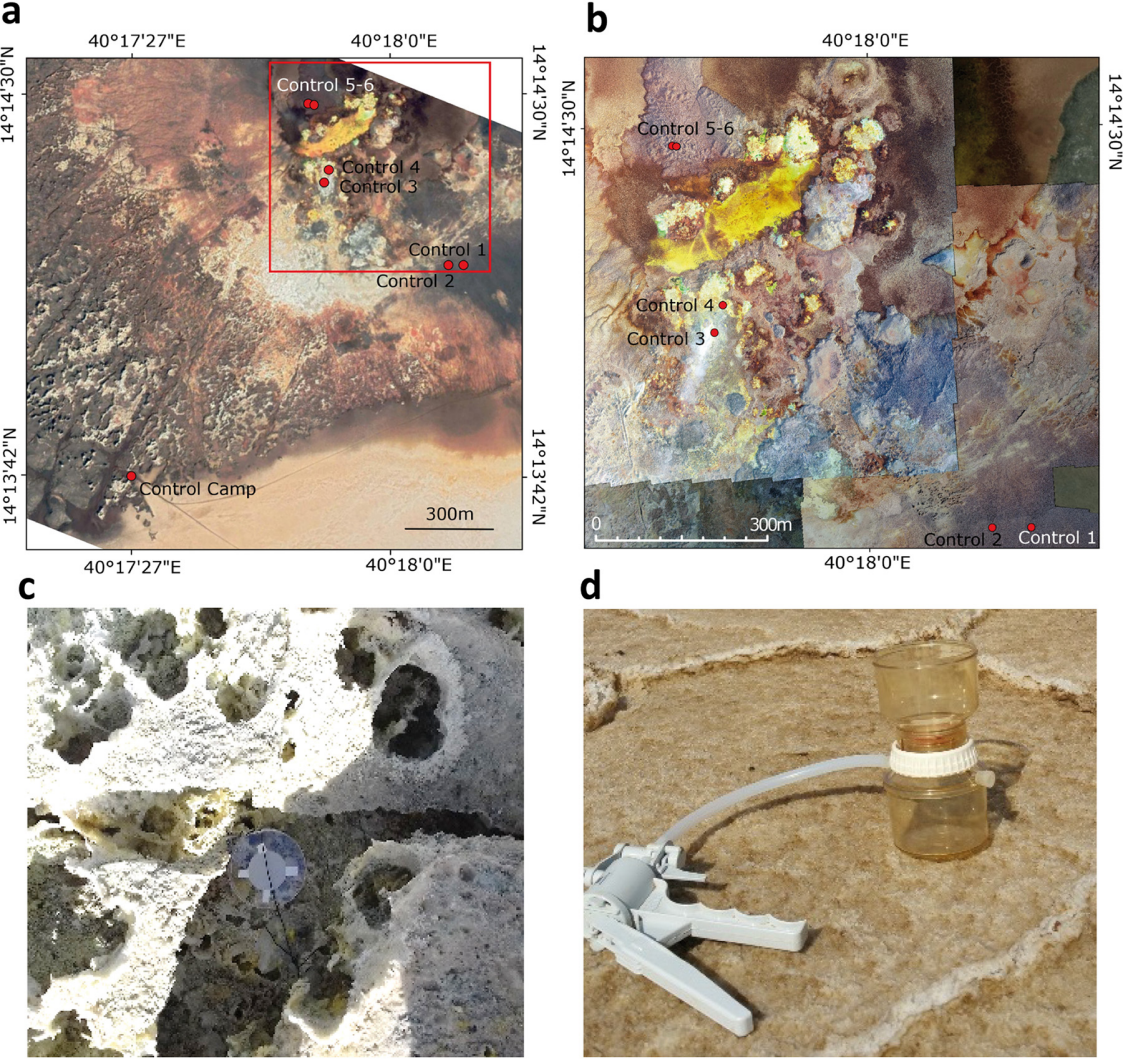
Location of aerosol sample collection sites and devices used on the Dallol dome and salt canyons. (a) Sampling sites of georeferenced control aerosol samples (Google satellite picture from 26 February 2019). (b) Detail of the dome sampling sites in assembled aerial Phantom-4 drone images (©Olivier Grunewald, used with permission). (c) Passive collecting system (here, filter 4 on a petri dish). (d) Active collecting system (filtration device connected to a manual pump) at the camp site.

To study the diversity of airborne microorganisms, we exposed to the open air six filters of 4.7-cm diameter and 0.2-μm pore size at different sites on top of the Dallol dome (Fig. 1; Fig. S1; Table S1) for 5 days. In addition, we manually filtered ∼2 to 3 m^3^ air through another filter, which was subsequently left exposed to the atmosphere for 11 days, at the southwest rim of the Dallol dome (camp site; Ctrl-Camp). Since we did not expect high biomass on the filters exposed on the dome (total surface, ∼100 cm^2^), we pooled DNA extracted from the six filters in a single final purification step (Ctrl-Dome; see Materials and Methods). We amplified 16S and 18S rRNA gene fragments from the two aerosol samples (Ctrl-Dome and Ctrl-Camp) using prokaryote- and eukaryote-specific primers. To our surprise and unlike results with previously analyzed Dallol brine samples (48), PCRs readily yielded amplicons, suggesting that considerable amounts of cells had accumulated on the filters.

10.1128/mbio.00307-22.1 TABLE S1 List of samples from the Dallol area featured in this study. Download Table S1, PDF file, 0.05 MB. Copyright © 2022 Belilla et al. 2022 Belilla et al. https://creativecommons.org/licenses/by/4.0/ This content is distributed under the terms of the Creative Commons Attribution 4.0 International license.

10.1128/mbio.00307-22.3 FIG S1 Areas of aerosol collection on the geothermal Dallol dome and dust accumulation in the chocolate formation. (a) Overview of the ancient hydrothermal chimney area close to the tourist path chosen to expose filters 1 and 2. (b) Filters 1 and 2 were exposed on top on two extinct chimneys, hidden from tourist sight. (c) Filter 2 in place. (d and e) Drone image of the hottest active degassing area (45 to 60°C air temperature) chosen to place filter 3. (f) Lateral view of the active degassing area. (g) Placing filter 3 for exposure. (h) Placing filter 4 for exposure ∼30 m northeast of filter 3, between salt boulders. (i) Filter 5 exposed on the chocolate formation on top of the dome. (j) Filter 6, exposed a few meters away from filter 5 in the same chocolate formation. (k) Photographs of the chocolate formation taken in January 2016, after a rain episode (left), and in January 2017. Note the whitish-yellowish layer of dust deposited by the wind. Download FIG S1, PDF file, 1.7 MB. Copyright © 2022 Belilla et al. 2022 Belilla et al. https://creativecommons.org/licenses/by/4.0/ This content is distributed under the terms of the Creative Commons Attribution 4.0 International license.

**FIG 2:**
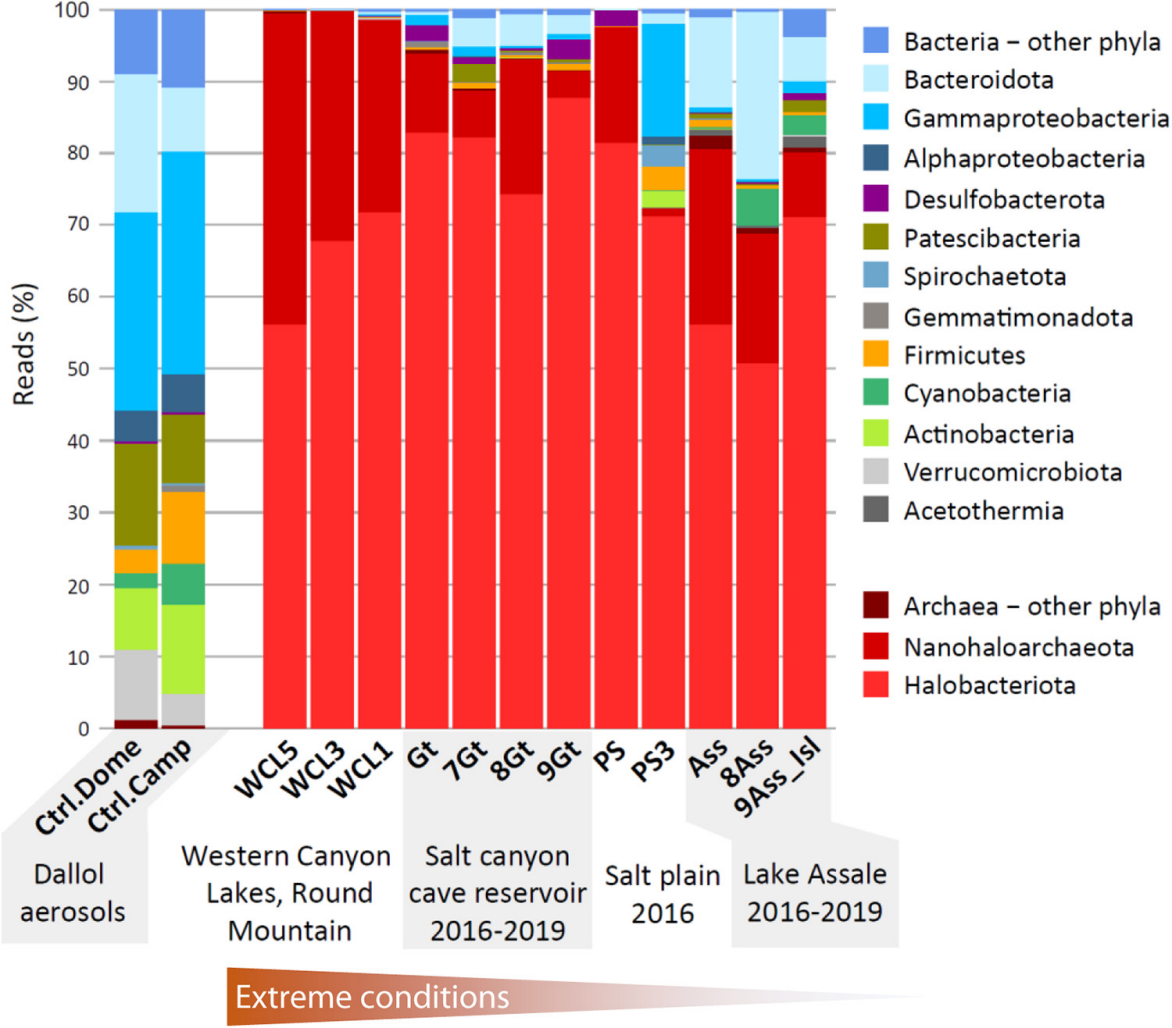
Histogram showing the diversity and relative abundance of 16S rRNA amplicon sequence reads in aerosols compared to autochthonous prokaryotic communities in the Dallol area, north Danakil. Brine samples are ordered following a gradient of extreme conditions, with the Western Canyon Lakes to the west of the Dallol dome displaying the most extreme conditions.

We then analyzed the diversity of Illumina-generated sequences including, for comparison, sequences from surrounding ecosystems produced in this and previous studies (Table S1 and S2). They consisted of samples from the salt plain at the dome base (PS samples), three Western Canyon Lakes (WCL samples) and samples from a cave reservoir in the Dallol west salt canyons (Gt samples) and from Lake Assale (Ass samples) collected in different years (2016 to 2019). Aerosols exhibited a wide prokaryotic diversity, but the global pattern was very different from that displayed by neighboring hypersaline ecosystems. Aerosols were largely dominated by bacteria (98 to 99%), whereas surrounding ecosystems bore much greater archaeal abundances (∼70 to 99%), with more extreme settings exhibiting higher archaeal proportions, notably Gt samples (88 to 95%), PS (97%) and the WCLs (99%) (Fig. 2). A large fraction of airborne bacteria affiliated with groups common in soil environments, such as *Acidobacteria*, *Verrucomicrobia*, *Bacteroidetes*, *Proteobacteria*, and *Actinobacteria* (58), which might disperse from the nearby Ethiopian mountains to the west and the Danakil block in the east Eritrean side. However, compared to typical soils, we observed increased proportions of *Gammaproteobacteria* (especially *Burkholderiales* and *Pseudomonadales*) (Table S2B), *Actinobacteria*, and *Firmicutes*, which, consistent with their higher resistance to desiccation and UV, are typically found in aerosol dust (27, 28, 31, 37).

10.1128/mbio.00307-22.2 TABLE S2 Sequence statistics and taxonomic assignation of gene amplicon sequences. (A) Sequence statistics and alpha diversity parameters. (B) Taxonomic assignation and abundance of prokaryotic 16S rRNA gene amplicon sequences (ASVs/OTUs) in Danakil Depression brines. (C) Taxonomic assignments and abundance of eukaryotic 18S rRNA gene amplicon sequences (ASVs/OTUs) in Danakil Depression brines. (D) Taxonomic assignation and abundance of prokaryotic 16S rRNA gene amplicon sequences (ASVs/OTUs) in the PS sample maintained in mesocosm in the laboratory since 2016, used for fluorescent staining and FISH experiments. Download Table S2, XLSX file, 1.3 MB. Copyright © 2022 Belilla et al. 2022 Belilla et al. https://creativecommons.org/licenses/by/4.0/ This content is distributed under the terms of the Creative Commons Attribution 4.0 International license.

Likewise, we detected a wide diversity of eukaryotes in Dallol aerosols (Fig. S2). Many of them were also affiliated with clades abundantly detected in soils, such as *Fungi, Cercozoa*, and *Alveolata* (mostly *Ciliophora* and *Apicomplexa*) (58), but others likely had a marine origin and might have come from the nearby Red Sea. In particular, we detected relatively large amounts of diatom (Ochrophyta) sequences, confirming their aptitude to disperse with the wind (33), but also cryptophytes and even a few radiolarian (*Acantharia*) sequences (Table S2C). The load of eukaryotes in aerosols seemed to be proportionally higher than in some local extreme ecosystems, as we were unable to amplify eukaryotes from the selected WCLs as well as, in some years, from Gt samples. The latter was possibly due to differences in the cave reservoir level affecting physicochemical parameters and/or in lower sampled volumes (sampled volumes were 5 to 25 L, but they were lower in Gt 2016 and PS samples [48, 51]). In the case of Gt 2016 and PS3, where fungal and choanoflagellate sequences appeared dominant in proportion (Fig. S2), the number of identified eukaryotic OTUs was very low (5 and 2, respectively; Table S2A), suggesting airborne contamination and illustrating the relevance of stochastic effects on low-biomass metabarcoding analyses (18).

10.1128/mbio.00307-22.4 FIG S2 Presence, diversity, and relative abundance of eukaryotic 18S rRNA gene amplicon sequences detected in Dallol bioaerosols compared with life-hosting environments in the Dallol surroundings. Empty boxes correspond to samples for which amplicon sequences could not be obtained. Assignation and frequency of the different ASVs/OTUs are given in Table S4. 8Gt and 8Gt-MB3, 8Ass and 8Ass-MB3, and 9Ass-Isl and 9Ass-Isl-CT are replicates sequenced in independent Illumina runs. Note that eukaryotes are detected only sporadically in Gt samples from the cave reservoir depending on the year and the fluctuating level (and associated conditions) of the brine. Note also that in Gt and PS3 samples, the number of ASVs was extremely small (5 and 2, respectively; see Table S2), notably making the opisthokont sequences in these two samples suspect for exogenous contamination (these ASVs were also found in aerosols). Download FIG S2, PDF file, 0.3 MB. Copyright © 2022 Belilla et al. 2022 Belilla et al. https://creativecommons.org/licenses/by/4.0/ This content is distributed under the terms of the Creative Commons Attribution 4.0 International license.

**FIG 3:**
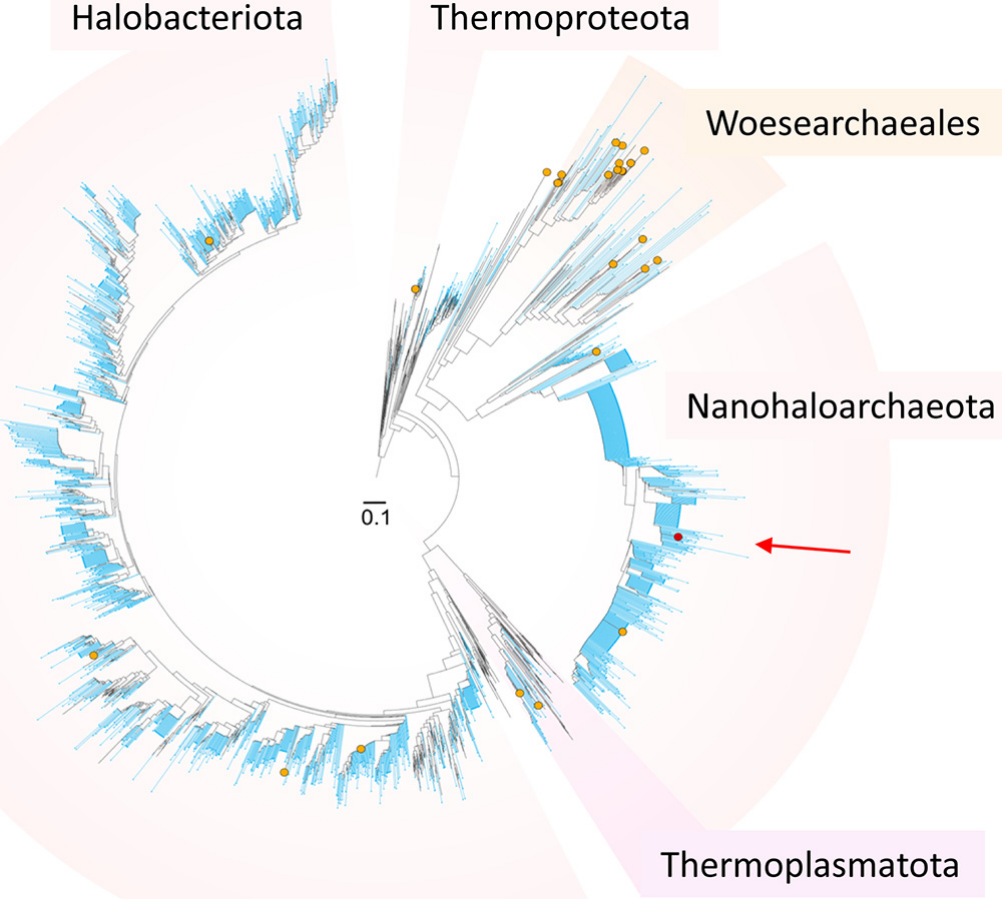
Phylogenetic tree of archaeal 16S rRNA gene sequences showing the phylogenetic placement of archaeal OTUs detected in Dallol aerosols as well as from systems unambiguously harboring microbial communities around the Dallol dome. OTUs from aerosol samples are indicated by orange dots. Blue lines indicated sequences previously determined from Lake Assale/Karum (Ass samples), a cave hypersaline reservoir at the salt canyons (Gt samples), and the salt plain at the dome base (PS samples) (48). The red dot (arrow) corresponds to the single OTU identified on a Dallol salt chimney wall by Gómez et al. (57). This OTU is nested among almost identical OTUs found in Ass, Gt, and PS samples (see a more detailed tree in Fig. S3). Reference sequences and best hits in GenBank are shown with black lines.

In addition to microorganisms typical of soil, airborne dust particles and seawater, we also detected in the Dallol aerosols sequences of lineages specifically found in Danakil extreme ecosystems, albeit in very minor proportions. We identified 1 to 2% archaeal sequences. Archaeal amplicon sequence variants (ASVs; we indistinctly refer to them as OTUs hereafter) affiliating with *Woesearchaeales* were relatively diverse; they might have a foreign origin, since they were present but proportionally less abundant in Danakil ecosystems (48). However, we also detected several haloarchaeal and nanohaloarchaeal ASVs that were very closely related to previously identified archaeal sequences in Ass, Gt and PS samples (Fig. 3). Furthermore, as shown in a molecular phylogenetic tree, the single OTU identified on a Dallol chimney wall by Gómez et al. (57) was closely related to OTUs from Dallol neighboring ecosystems (Fig. 3; Fig. S3, red arrow). Incidentally, some of these PS, Ass, and Gt nanohaloarchaeal OTUs closely related to the sequence reported by Gómez et al. (57) were also previously detected sporadically in samples of Dallol brines (e.g., DAL6A) and the Yellow Lake (YL) (Fig. S3) but only after reamplification by nested PCR (and with “negative” controls yielding amplicons) and in a background of largely dominant (>98 to 99%) contaminant bacterial sequences, which suggested that they were dispersing forms or undesired contaminants during sample processing in the field or in the laboratory (48). Our results indicate that aerosols from the Danakil desert are vehicles for the dispersal of extremophiles from local hypersaline ecosystems and might constitute a source of difficult-to-discern contaminants in nearby settings not permissive for active life.

10.1128/mbio.00307-22.5 FIG S3 16S rRNA phylogenetic tree of *Archaea* showing the position of OTUs identified in polyextreme environments at and surrounding the geothermal dome of Dallol (Danakil Depression, Ethiopia). OTUs identified in bioaerosols either at the camp site at the salt canyons (Ctrol_Camp) or on top of the dome (Ctrol_dome) are shown in blue. The nanohaloarchaeal sequence from the only OTU identified by Gómez et al. (57) on a small chimney wall on the Dallol dome is indicated in red (arrow). The names of OTUs indicate the samples they were identified in. Most samples contain diverse microbial communities: Ass, AssPJ, Lake Assale; Gt, 7Gt, 7Gt-pp, cave reservoir at the Dallol salt canyons; PS, PS3, salt plain at the base of the Dallol dome. A few samples might contain incipient aerosol-transported communities forming an incipient soil: 7YLs, 7YL-s, soil-like crusts around Yellow Lake; 7DA5s, sediment-like deposit at the dome. OTUs rarely detected in a few Dallol or Yellow Lake samples always correspond to abundant OTUs detected in life-containing systems in the Dallol area and were considered contaminants: YL2, 7YL Yellow Lake; DAL6A7DA10, 7DA14, Dallol dome (48). Bootstrap values higher than 50% are indicated at nodes. Some clades without Dallol sequences were collapsed. Download FIG S3, PDF file, 0.9 MB. Copyright © 2022 Belilla et al. 2022 Belilla et al. https://creativecommons.org/licenses/by/4.0/ This content is distributed under the terms of the Creative Commons Attribution 4.0 International license.

### Cells and DNA are almost instantly degraded in hyperacidic Dallol brine.

**FIG 4:**
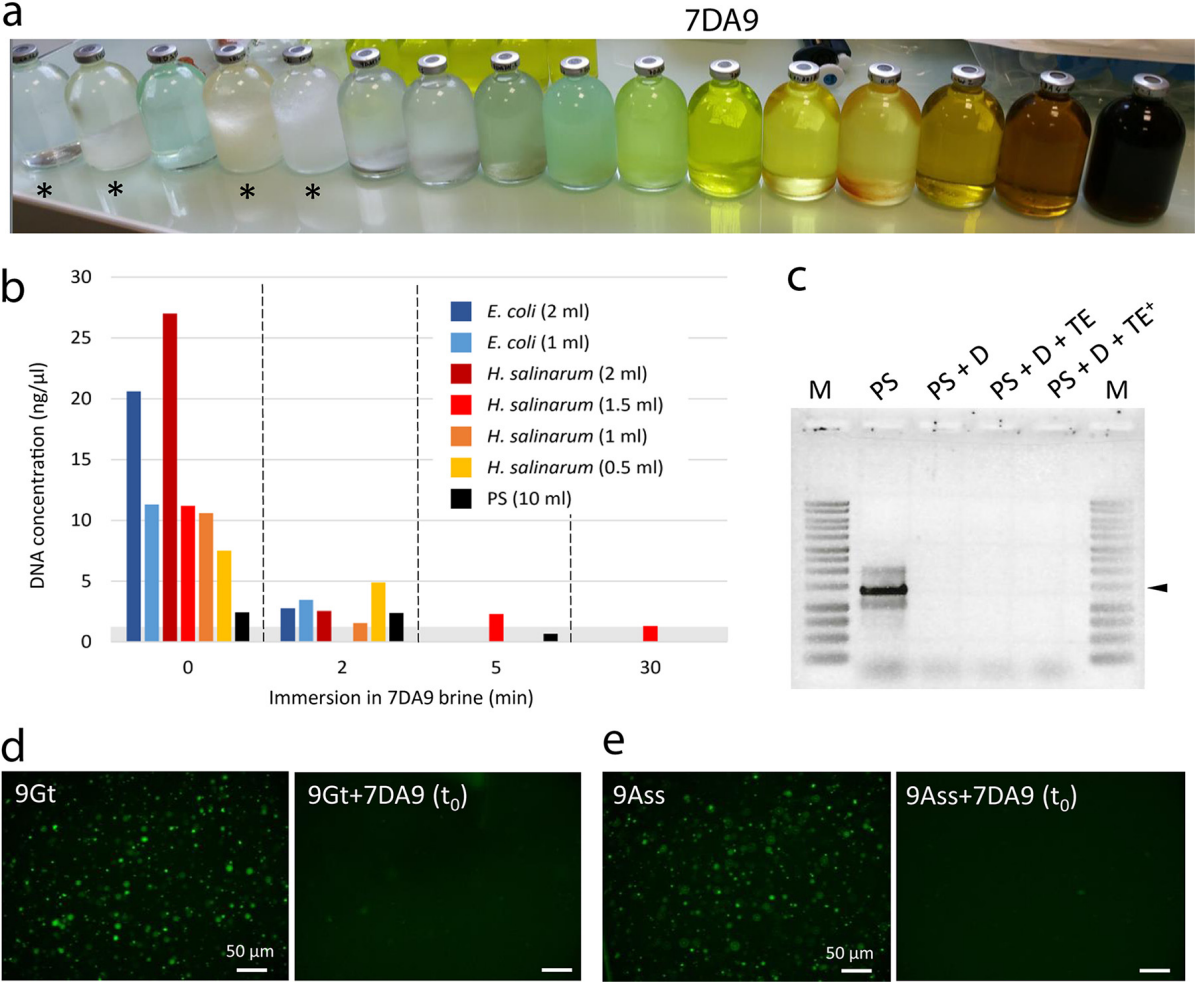
Cell- and DNA-degrading effects of hyperacidic Dallol brines. (a) Different samples of Dallol brines collected in 2017 (except for samples labeled with an asterisk, corresponding, from left to right, to the cave reservoir, Yellow Lake, and Black Lake [two samples of each]). The different colors reflect the origin of samples along physicochemical gradients. The more transparent and light translucent green samples correspond to hot (∼108°C) acidic (pH ∼0) brines springing from salt chimneys. As brines cool down, they get progressively oxidized and concentrate, reaching pH values as low as −1.5/−1.7 (48, 50). Sample 7DA9, used for these experiments, is indicated. (b) DNA degradation caused by the immersion of different amounts of cells from E. coli and *H. salinarum* cultures and a mesocosm-maintained salt plain community (PS) in 7DA9 brine for increasing amounts of time (see the text). The gray rectangle indicates the detection limit of measurements. (c) 16S rRNA gene amplifying test on resuspended cell pellets from the PS sample (maintained in laboratory mesocosm) after contact with acidified (pH ∼0) hypersaline medium (solution D) mimicking Dallol brines. Contact with solution D lasted around 11 min (vortexing followed by 10 min centrifugation) in PS + D but less than 30 s in samples neutralized with TE or TE+ solutions (see the text). Pellets were resuspended in 10 mM Tris (pH 8.5) prior to PCR amplification. M, size markers; the arrowhead indicates 1,500 bp. (d and e) SYTO 9-induced epifluorescence of 9Gt and 9Ass cells, respectively, before and immediately after (t_0_) adding 2 volumes of 7DA9 brine. Similar effects can be seen in other samples (Fig. S4).

Airborne particles deposited on a rather small surface (∼100 cm^2^; Ctrl-Dome) for only 5 days readily yielded a wide diversity of prokaryotic and eukaryotic amplicons. This implies that a relatively abundant dust-associated incoming biomass seeds the Dallol brines over time, transported by the strong local winds (42, 43). Indeed, between 2016 (after a rain, washing episode) and 2017, a conspicuous layer of whitish-yellowish dust had visibly covered the dark “chocolate” formation (40, 50) (Fig. S1k). Then, why was it virtually impossible to amplify rRNA genes from Dallol ponds even after the filtration of many liters of brine (48)? We hypothesized that Dallol hyperacidic brines not only do not harbor life but also continuously destroy the incoming cells and DNA owing to their polyextreme chemistry. To test this idea, we studied the effect of Dallol hyperacidic brines on cultures of the bacterium Escherichia coli and the haloarchaeon Halobacterium salinarum, as well as on cells from natural communities of Dallol neighboring ecosystems. We selected brine 7DA9 to carry out these experiments. It represented an intermediate stage along the oxidation and acidification gradient observed *in situ* (Fig. 4a) (48, 50), with the fluid having cooled down to local ambient temperature (pH −0.34, 31.9°C, 38% salts) (Table S1).

10.1128/mbio.00307-22.6 FIG S4 Additional examples of cell and DNA-degrading effects of hyperacidic Dallol brines. (a) 16S rRNA gene-amplifying test on resuspended cell pellets from natural samples and cultures after contact with acidified (pH ∼0) hypersaline medium (D) mimicking Dallol brines. From left to right, panels correspond to treatment of cells from the Lake Assale (8Ass), the cave reservoir at the salt canyons (Gt), Halobacterium salinarum, and positive and negative controls of the experiment. Contact with D lasted around 11 min (vortexing followed by 10 min centrifugation) in samples + D but less than 30 s in samples neutralized with TE or TE+ solutions (see the text). Pellets were resuspended in 10 mM Tris (pH 8.5) prior to PCR amplification. M, size markers. (b) SYTO 9-induced epifluorescence of various brines at the Dallol dome and surrounding systems before and immediately after (t0) adding 2 volumes of 7DA9 brine. 9Ass, Lake Assale (2019); 8Ass, Lake Assale (2018); 9Gt, cave reservoir (2019); 8Gt, cave reservoir (2018); PS, salt plain; 7DA9, Dallol pond hyperacidic brine (2017). Bar, 50 μm. Download FIG S4, PDF file, 0.7 MB. Copyright © 2022 Belilla et al. 2022 Belilla et al. https://creativecommons.org/licenses/by/4.0/ This content is distributed under the terms of the Creative Commons Attribution 4.0 International license.

First, cells from different volumes (0.5 to 2 mL) of the same E. coli and *H. salinarum* cultures were retained on 0.2-μm-pore-size filters (2.5-cm filter diameter) using a small vacuum filtration device prior to the addition of 500 μL of 7DA9 brine. We treated the cells from 10 mL of a PS sample maintained in a laboratory mesocosm under salt-saturating conditions. Immediately after the desired incubation time (2, 5, and 30 min), the 7DA9 brine was vacuum filtered away and the cells were rinsed with 2 mL of Milli-Q water. DNA was subsequently purified from filters and quantified with Qubit high-sensitivity assays. As can be seen in Fig. 4b, after 2 min of incubation DNA concentration drastically diminished, and after 5 to 30 min, no measurable DNA above the error limit of the apparatus could be detected. To eliminate the possibility that trace DNA amounts remained, we carried out 16S rRNA gene PCR amplification, which failed in all cases. To see if these effects could be reproduced with a solution mimicking Dallol brine chemistry, we attempted to amplify 16S rRNA genes from cultures (E. coli and *H. salinarum*) and natural PS, Gt, and Ass samples immediately after contact with an artificial brine (D, pH ≤ 0). Cell pellets from identical sample volumes were resuspended in D and either directly centrifuged in the brine for 10 min or neutralized with TE and TE+ solutions (see Materials and Methods) prior to centrifugation. After applying DNA purification protocols, no 16S rRNA gene amplification could be observed in any of the samples tested (Fig. 4c and Fig. S4a). Finally, we also aimed at directly looking at the effect of Dallol brines on cells. For this, we observed cells from different natural hypersaline systems around Dallol (Gt, Ass and PS samples) stained with the DNA fluorescent dye SYTO 9 before and immediately after contact with 7DA9 brine. In all cases, cell-associated fluorescence completely disappeared (Fig. 4d; Fig. S4b). However, in some microscopy fields fluorescent particles occasionally remained visible (e.g., 9Ass and PS panels in Fig. S4b). They often had irregular shapes and were most likely mineral precipitates.

Collectively, these experiments strongly suggest that airborne dispersing cells continuously falling on the Dallol hyperacidic brines, including those from neighboring hypersaline ecosystems, are rapidly destroyed upon arrival. From this perspective, it is interesting that in their study, Gómez et al. could apparently amplify 16S rRNA genes only from a salt chimney wall (D9) and not from a neighboring hyperacidic brine (D10). We interpret this brine as hyperacidic, with a pH of ≤0, although they reported a pH of 2.42, higher than the pH of 0.25 measured in their chimney D9 hot brine (57). However, other studies have recurrently measured pHs of ≤0 in the dome brines (48, 50). The yellow color of the D10 pond (Figure 1 in reference 57) indicates a mix of reduced Fe (transparent to light greenish color) and oxidized iron hydroxo- and chloro- complexes (yellow to ochre colors in the Dallol brines; see the color progression in Fig. 4a), which is concomitant with increased oxidation and acidification going from pH ∼0 to pH values as low as −1.6 (48, 50). Nonetheless, although they were unable to amplify 16S rRNA genes from their pond D10 sample, Gómez et al. (57) did detect potential cells by FISH in that brine. However, our observations of samples stained by fluorescent SYTO 9 (Fig. S4b) suggested that mineral particles could retain this dye. Likewise, previous flow cytometer cell sorting (FACS) analyses of Dallol brines stained with the fluorescent dye DRAQ5 followed by scanning electron microscopy (SEM) observations clearly showed that halite crystals were autofluorescent and/or fluorescently labeled and could be sorted by FACS on the basis of it (48). Accordingly, could local mineral precipitates constitute a confounding factor for detection of life by fluorescent staining and FISH in Dallol brines?

### Nonspecific binding of fluorescent dyes and oligonucleotide probes to mineral precipitates in Dallol brines.

**FIG 5:**
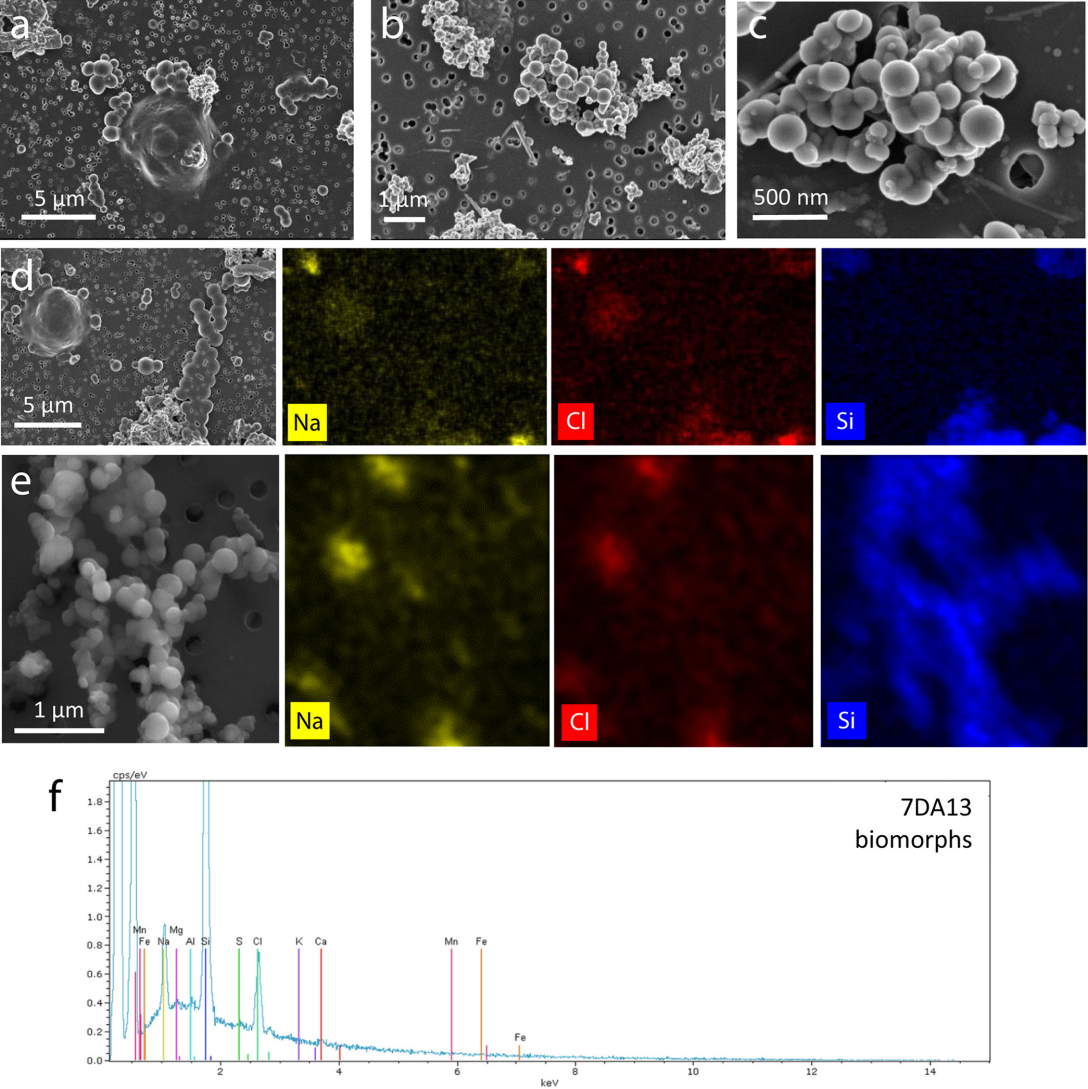
SEM and EDXS chemical mapping of salt plain (PS) cells and Dallol brine mineral biomorphs. (a) PS nanosized cells surrounding a bigger cell (see epifluorescence images of PS cells also in Fig. 6). (b) Nanosized biomorphs from Dallol 7DA13 hyperacidic brine. (c) Close view of Dallol brine DAL4 nanosized biomorphs. (d) Cells of diverse sizes from the PS sample and associated EDXS chemical maps (right); note some nanosized cells associated with a larger coccoid cell (upper left of SEM picture). (e) SEM image of coccus-like silicate biomorphs from Dallol 7DA13 and associated chemical maps (right). Color intensity provides semiquantitative information of the mapped elements. (f) EDXS spectrum of 7DA13 brine biomorphs.

To test whether mineral particles in Dallol and surrounding hypersaline brines can nonspecifically adsorb fluorescent dyes, we first looked for the presence of biomorphs and more irregular mineral precipitates in the samples that we used for subsequent FISH experiments. Previous SEM analyses coupled with energy-dispersive X-ray spectrometry (EDXS) indeed showed the occurrence of abiotic particles resembling cells, mostly silica and sulfur based, in Dallol and other Danakil brines (48, 51). These observations were consistent with a wide morphological diversity of crystals from the macro- down to the nanoscale (50). Many crystalline morphologies were, however, interpreted as microfossils by Gómez et al. (57) without considering plausible alternative explanations. Here, as in previous studies (48), we aimed at comparing natural samples unambiguously containing microbial communities with the Dallol brines. For our experiments, we used two Dallol brines (7DA13 and DAL4) (Table S1) and, as a positive cell-bearing control, the PS sample originally collected in 2016 that was slightly diluted and maintained under salt-saturating conditions in the laboratory. This sample was originally largely enriched in *Halobacteriota* and *Nanohaloarchaeota* (Fig. 2) and, although upon laboratory incubation its community composition evolved, members of both taxa were well represented in the sample at the time when we carried out these experiments (Fig. S5; Table S2D). Confirming previous studies (48), SEM and EDXS showed the presence of mineral biomorphs in all the samples, but we could observe unambiguous cells in PS only (Fig. 5; Fig. S6). Many of these cells were very small and often attached to larger, smoother-looking cells, suggesting that they could be members of the epibiotic *Nanohaloarchaeota* attached to haloarchaeal cells (Fig. 5a and d). Rounded mineral precipitates (biomorphs) resembling cells tended to be smaller than *Nanohaloarchaeota*-like cells (Fig. 5b and c; Fig. S6). However, they could be clearly distinguished by EDXS, since biomorphs were silica rich (Fig. 5e and f; Fig. S6s to u), whereas mineral-forming elements (e.g., Cl, Na, and Si) could not be spotted on cells in significant amounts (Fig. 5d; note, however, the presence of silica-rich biomorphs in the lower part of the panel, at the base of the cell agglomerate). In addition to biomorphs, other mineral precipitates with varied shapes and elemental compositions were observable (Fig. S6).

10.1128/mbio.00307-22.7 FIG S5 16S rRNA gene-based prokaryotic diversity present in the PS sample maintained in a laboratory mesocosm at the time when FISH experiments were carried out. The upper panel corresponds to dominant taxa; the panels below provide details about the diversity of less abundant taxa. Download FIG S5, PDF file, 0.5 MB. Copyright © 2022 Belilla et al. 2022 Belilla et al. https://creativecommons.org/licenses/by/4.0/ This content is distributed under the terms of the Creative Commons Attribution 4.0 International license.

10.1128/mbio.00307-22.8 FIG S6 Scanning electron microscopy images of cells and biomorphs from brines at the Dallol dome and the salt plain and elemental chemical maps and EDXS spectra of biomorphs and other mineral precipitates in the Dallol hyperacidic used in this study. Samples from the salt plain (PS; panels a to f) and from the Dallol dome hyperacidic brines 7DA13 (g to l) and DAL4 (m to r). Samples with chemical maps correspond to 7DA13 (s) and DAL4 (t to u). SEM photographs were taken using In Lens (t) or AsB detectors. Download FIG S6, PDF file, 2.2 MB. Copyright © 2022 Belilla et al. 2022 Belilla et al. https://creativecommons.org/licenses/by/4.0/ This content is distributed under the terms of the Creative Commons Attribution 4.0 International license.

**FIG 6:**
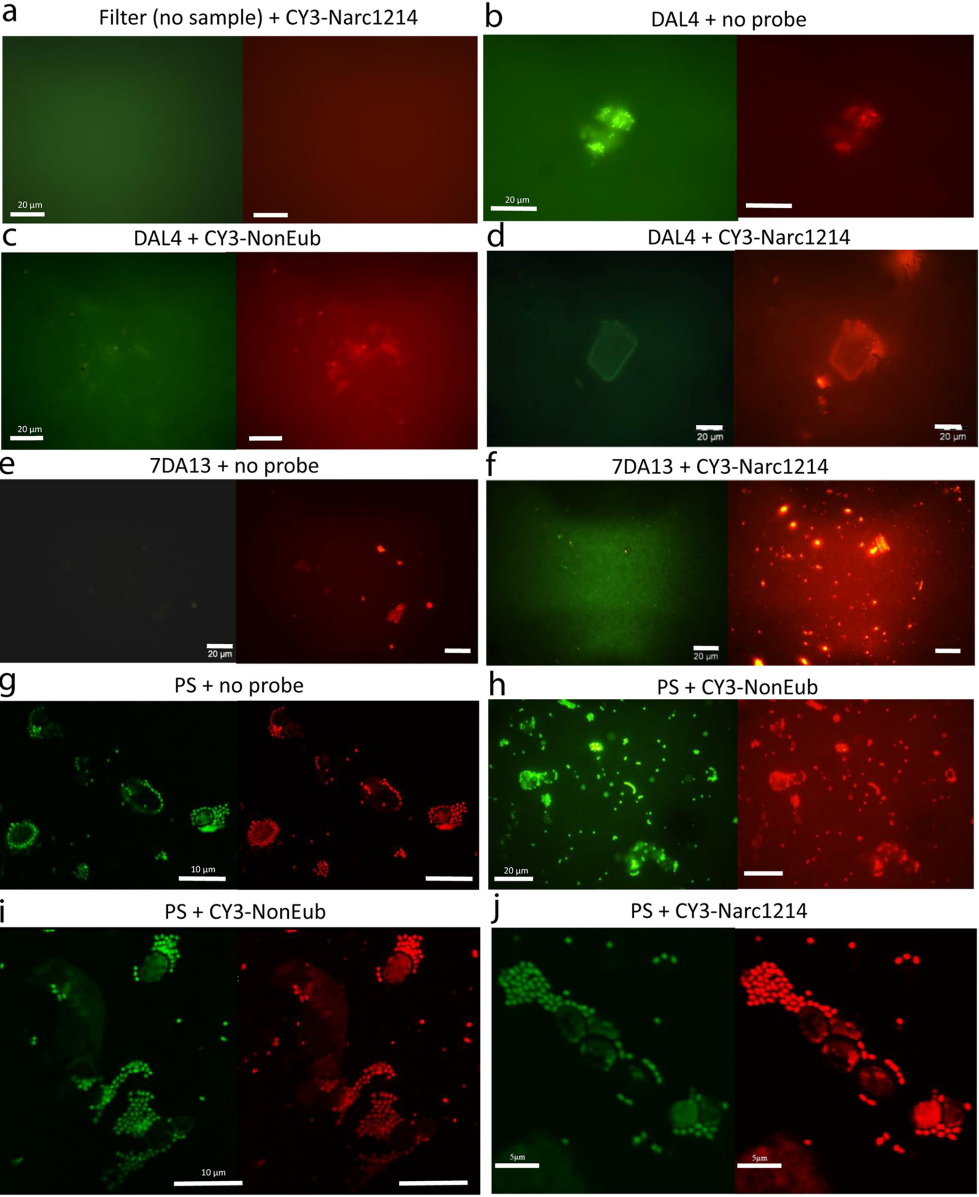
Fluorescent staining and FISH experiments on Dallol hyperacidic brines (DA4 and 7DA13) and the salt plain (PS) ecosystem. In all cases, the DNA-intercalating agent SYBR Gold was used for fluorescent staining (green, left panels). The red fluorescence of CY3-labeled probes (or autofluorescence at the same wavelength) is shown in the right panels. The names of the samples and the probes used are indicated. (a) Autofluorescence control of empty 0.22-μm-pore-size filters used to retain cells in subsequent experiments. (b to f) Dallol brines after FISH hybridization without probe, with the nonspecific probe CY3-NonEub and the *Nanohaloarchaeota*-specific probe Narc1214. (g to j) FISH experiments, including controls, of the PS samples. Images a to f and h were obtained by epifluorescence microscopy; images g, i, and j are CLSM images. FISH hybridization conditions were the same as those described in reference 57.

We subsequently tested whether fluorescent staining and FISH probes could bind mineral particles nonspecifically and could have been a confounding factor in previous analyses drawing conclusions about the presence of life in Dallol brines (57). For this purpose, we carried out FISH experiments in our samples using the same probes and experimental conditions as described by Gómez et al. (57) but adding controls that were missing in their analysis. We thus used the DNA-intercalating agent SYBR Gold (green fluorescence) and the CY3-labeled *Nanohaloarchaeota*-specific probe Narc1214 (red fluorescence) as described previously (57). Additionally, we included FISH controls with no probe or with the widely used control probe NonEub (Non-Bact338) to exclude unspecific probe binding (59). In general, we observed a strong red autofluorescence of both cells and mineral particles under the assayed conditions (Fig. 6; Fig. S7). Thus, although control filters without any sample did not show particle autofluorescence upon FISH (Fig. 6a), Dallol brines exhibited autofluorescent particles in several microscopy fields after FISH in the absence of probe or after hybridization with NonEub (Fig. 6b, c, and e). In addition, upon hybridization with Narc1214, more or less irregular particles were also labeled (Fig. 6d and f; Fig. S1a, b, g, and h). Those particles most likely corresponded to mineral precipitates, since the morphology of fluorescently labeled PS cells, including probable tiny *Nanohaloarchaeota*, was very different (Fig. 6g to j; Fig. S7c to e). The latter appeared indeed as very small, intensely labeled cells most often surrounding larger cells, possibly haloarchaea, exhibiting less intense fluorescence. Larger irregular particles showing fluorescence were also observed in several PS fields (e.g., Fig. 6i and j [lower left quadrant] and Fig. S7f). However, under the assayed conditions (57), FISH hybridization was not specific, since cells exhibited similar fluorescence without probe and after hybridization with the probes NonEub and Narc1214. Images of FISH experiments with different probes (Cy3-ARC915 and EURY498-FITC) and DAPI (4′,6-diamidino-2-phenylindole) as the nucleic acid dye also showed some unspecific fluorescence (Fig. S8, note that images were taken at the same light intensity). Upon image treatment or depending on the confocal laser scanning microscopy (CLSM) mode (sequential or not) (Fig. S7g and h), low (auto)fluorescence signals can be increased to levels that may appear significant. Collectively, our experiments show similar unspecific binding of SYBR Gold and CY3-labeled probes to mineral precipitates and cells under the assayed conditions. Nonetheless, cells, when unambiguously present (PS), displayed a morphology significantly different from that of mineral particles.

10.1128/mbio.00307-22.9 FIG S7 Additional images from FISH experiments on Dallol hyperacidic brines and the salt plain ecosystem using the *Nanohaloarchaeota*-specific probe CY3-Narc1214. In all cases, the DNA-intercalating agent SYBR Gold was used for fluorescent staining (green, left panels). The red fluorescence of CY3-Narc1214 is shown in the right panels. The names of the samples and the probes used are indicated. (a and b) Epifluorescence microscopy of FISH experiments on Dallol brine 7DA13. (c to f) epifluorescence microscopy of FISH experiments on salt plain (PS) sample. (g) CLSM images of FISH hybridization experiments on 7DA13 brine. (Left) Superposition of SYBR Gold, DAPI, and CY3-Narc1214 fluorescence (note the high background due to unspecific staining of the salt embedded filter). (Right) Magnification of the area framed in red and CY3-Narc1214 binding to mineral particles after increasing contrast. (h) CLSM images of a FISH hybridization experiment on DAL4 brine. Left and right panels show superposition of the SYBR Gold, DAPI, and CY3 fluorescence, but the left panel was obtained in sequential mode. FISH hybridization conditions were the same as described by Gómez et al (57). Download FIG S7, PDF file, 0.8 MB. Copyright © 2022 Belilla et al. 2022 Belilla et al. https://creativecommons.org/licenses/by/4.0/ This content is distributed under the terms of the Creative Commons Attribution 4.0 International license.

10.1128/mbio.00307-22.10 FIG S8 Comparative epifluorescence microscopy of salt plain (PS) mesocosm samples and Dallol 7DA13 hyperacidic brines in FISH experiments using control and archaeon-specific probes. Hybridized samples were observed by epifluorescence microscopy (Zeiss Axioplan) using filters for the detection of Cy3 (left panels), FITC (central panels) and DAPI (right panels). Probes labeled with FITC and Cy3 are indicated in green and orange, respectively. DAPI staining is indicated in blue. For proper comparison, the light intensity at which all micrographs were taken was identical. (a to f) Control FISH experiments on PS microbial cells hybridized with no probes (a to c) or with the control probe nonBAC388 (complementary to a universal bacterial probe) (d to f). Note the slight natural autofluorescence of cells observed with the FITC filter (b and e) and some autofluorescence of NonEub-hybridized cells with the Cy3 filter (d). (g to o) Samples hybridized with the archaeal probes ARC915 and EURY498. (g to i) Hybridization of 7DA13 brine. Most fields were fully dark, but occasionally, mineral particles adsorbed the probes (DAPI staining, low to none). (j to o) Hybridization of archaeal PS cells with ARC915 and EURY498. Note also the red autofluorescence of a green algal cell (n). Bars, 20 μm. Download FIG S8, PDF file, 0.6 MB. Copyright © 2022 Belilla et al. 2022 Belilla et al. https://creativecommons.org/licenses/by/4.0/ This content is distributed under the terms of the Creative Commons Attribution 4.0 International license.

The above observations imply that previous results based exclusively on FISH analyses without the use of appropriate controls and in the absence of complementary evidence are not conclusive. This is the case of the FISH study in a Dallol brine (D10) by Gómez et al. (57). Doubt can be also cast on another study claiming the extensive occurrence of methanogenesis at high temperature, high ionic strength, and pH close to zero in Dallol brines (60). In that study, the authors inoculated Dallol brines on culture media for methanogenic archaea and incubated these inoculated media at 45°C and 95°C for 6 months. Notwithstanding the fact that the culture medium was not hypersaline and had neutral pH (6.5 to 7.0) (60), the authors drew conclusions about thermoacidophilic methanogenesis at high ionic strength for two reasons. First, they observed the presence of CH_4_ in the headspace of bottles after 6 months of incubation. However, it is unlikely that putative methanogenic archaea adapted to extreme hypersaline and hyperacidic environments are active in low-salinity and neutral-pH media. In addition, the authors did not produce any 16S rRNA gene sequences or images of cells growing in culture. Furthermore, the δ-^13^C isotopic composition of CH_4_ between −37.0 and −32.6‰ observed by Sanz et al. (60) is more compatible with thermogenic (ca. −40‰) than biogenic (ca. −60‰) methane formation (61, 62). In addition, it is unclear whether CH_4_ was actually produced after 6 months or originally included with the brine inoculum, since CH_4_ was analyzed only at the end of the experiment and not at time zero. Therefore, in the absence of initial CH_4_ measurements (to see if thermogenic methane was present in the original sample) and of negative controls with no inoculum (to see if thermogenic methane is produced from the medium chemistry) and in the absence of gene or genome data demonstrating the occurrence of methanogenic archaea with typical adaptations of halophilic and acidophilic microbes, this result is far from conclusive.

Second, Sanz et al. performed FISH experiments on natural Dallol brine and salt samples using two CY3-labeled probes specific for, respectively, *Methanosarcinales* and *Methanobacteriales* and observed fluorescent signals (60). However, they did not include negative controls and, upon inspection of their FISH images, cellular morphology and size compatible with known methanogenic archaea are unclear (presence of irregular particles and some excessively tiny particles). Therefore, in the absence of adequate FISH controls and 16S rRNA gene sequences attesting to the presence of *Methanosarcinales* and *Methanobacteriales*, these results are ambiguous and may correspond to fluorescence artifacts. Furthermore, the presence of methanogens in surface brines subject to increased oxidation would be at odds with their strict anaerobiosis (they might, however, be present in high-temperature brines if the local extreme conditions were permissive for life). Indeed, sequences for members of these two orders have not been detected among the widely and dominant diverse archaea in life-hosting brines around the Dallol dome (Table S2B) (48, 51).

**Lessons for the unambiguous identification of cells in highly mineralized environments.** Determining the unambiguous occurrence of microbes thriving in highly mineralized polyextreme environments at the permissivity barrier for life poses challenges analogous to those associated with biosignature detection in the ancient fossil record (6) and extraterrestrial objects (7). Contamination from exogenous sources and the formation of abiotic structures with biogenic appearances constitute important confounding factors that can mislead conclusions. To avoid this, the implementation of strict and rigorous protocols with adequate controls is imperative. Under environmental conditions at the frontier between life and the mineral realm, the null hypothesis when searching for microorganisms or life traces should always be the absence of life. Proof for the occurrence of indigenous life should come in the form of irrefutable evidence, such as the isolation and cultivation of microbial species under physicochemical conditions mimicking the natural extreme environment. Alternatively, plausible life can be concluded from convergent observations coming from various sources that cannot be explained otherwise (i.e., by contamination or purely abiotic processes).

In this study, we have shown that airborne dispersal from distant sources (soil particles, marine aerosols) and local hypersaline ecosystems (Danakil salt desert brines) actively transport exogenous prokaryotic and eukaryotic microorganisms to the geothermal dome of Dallol. Most of the 16S rRNA gene sequences identified in aerosols corresponded to nonextremophilic bacteria but also to some halophilic archaea. The deposition of these contaminant extremophilic organisms on the Dallol salt formations can lead to erroneous inferences of in situ life development in the absence of controls and/or additional undisputable evidence. In addition to aerosols, another serious source of contamination is touristic activity, which has been continuously increasing in recent years (43, 63) until a recent armed conflict declared in the neighboring Tigray region. The Dallol dome is a relatively small mound (∼5 by 3 km), and the area on top displaying hydrothermal activity is much smaller. The daily arrival of dozens of tourists and their local guides climbing from the salt plain is a source of salt-loving (e.g., transported in shoes) and human-associated microbes that may be detected under forced nested-PCR conditions (48).

In this work, we also confirm the widespread occurrence of diverse mineral precipitates, including mostly silica-based biomorphs, in the hyperacidic hypersaline brines of Dallol. These mineral precipitates can unspecifically bind fluorescent nucleic acid dyes and oligonucleotide probes under FISH conditions previously used to infer the extensive presence of microorganisms in these brines (57, 60). Even if autofluorescence and unspecific probe binding can obscure the interpretation of FISH experiments, cell (notably *Nanohaloarchaeota*-like) morphologies can be clearly distinguished from mineral precipitates in natural microbial communities from the Dallol-adjacent salt plain (Fig. 6; Fig. S7). These results strongly suggest that previous observations of fluorescently stained structures (57, 60) likely corresponded to salt/mineral particles. Our study highlights the need to include controls and consider alternative abiotic explanations before safely drawing conclusions about the presence of life or biogenic signatures in extreme terrestrial or extraterrestrial systems.

Is there life in the hyperacidic and hypersaline Dallol brines? Considering the above observations and caveats, irrefutable evidence for microorganisms thriving in Dallol brines is still missing. On the contrary, our results, including experimental controls, strongly suggest the absence of life developing in Dallol brines and support the alternative explanation that putative archaea detected in these salts and brines are the product of nearby contamination and mineral particles binding fluorescent dyes and probes. Furthermore, we show that, upon arrival on Dallol brines, the DNA of exogenous microbial cells is rapidly destroyed, including that of cells from halophilic archaea and natural communities from neighboring hypersaline ecosystems (Fig. 4). This observation may hold some clue about why microbial life cannot cope with the local polyextreme conditions of the Dallol brine. Acid-induced depurination has long been known to be a cause of DNA degradation (64, 65). Despite this, extreme acidophiles are known to thrive down to an external pH of ∼0 (3), although the cytoplasmic pH is much higher (∼4.6) (66). Likewise, extremophilic organisms adapted to the individual parameters that reach extreme values for life in Dallol are known, such as extreme halophiles (4, 5) and hyperthermophiles (2) or some combinations of extremes (1), including low pH (≥1) and high salt concentrations (67–71).

Extremophilic organisms have also adapted to considerable concentrations of potentially harmful chemicals, such as reactive oxygen species (ROS) and perchlorates. Dallol aqueous chemistry is dominated by ferrous and ferric aqueous species (50) that are prone to generate ROS by homogeneous Fenton-like reactions (72). In addition, nanosized iron-oxyhydroxides, such as akageneite, goethite, and hematite, most abundant in Dallol pools (50), are known to initiate heterogeneous redox reactions resulting in ROS production (73). Some ROS are naturally produced during cellular redox processes, triggering oxidative damage in proteins, lipids, and nucleic acids that require active mechanisms of detoxification and repair (74, 75). The combined presence of large amounts of ROS in the surrounding medium and hypersalinity-hyperacidity might impose an even more serious challenge for life. Sodium perchlorate is a chaotropic salt primarily formed on Earth through the oxidation of atmospheric chlorine by ozone and/or oxygen-containing radicals found in the stratosphere, but it is also formed under high UV irradiation of NaCl in the presence of silica, conditions that are met on the surface of Mars (76). These conditions also occur in Dallol, often considered an analog of Mars (49), although perchlorates are likely much less abundant than the ferrous and ferric chloride complexes dominant in Dallol brines. Perchlorate is also an extreme oxidant with potential deleterious effects on cells. However, many halophilic archaea grow well in 0.4 M sodium perchlorate and can even use it as an electron acceptor (77). Some fungi can even tolerate 2.4 M perchlorate (78). Therefore, each of these physicochemical effectors is not necessarily lethal *per se*. However, the combination of these factors with a borderline water activity for life and a relatively high chaotropicity induced by the presence of high iron and calcium concentrations (Table S1) appears to be detrimental to life (48, 51, 56). The presence of chaotropic salts, perhaps combined with ROS, might impair membrane stability, facilitating the penetration of hyperacidic brine in cells that would lead to the rapid degradation of nucleic acids. Our results contribute to setting a barrier to life at the confluence of these polyextreme conditions combining hyperacidity, hypersalinity, ROS, and high abundance of chaotropic salts.

## MATERIALS AND METHODS

### Sample collection.

The list of samples from the Dallol dome and surrounding area used in this study as well as their collection dates and associated physicochemical parameters are provided in Table S1. Samples were collected in consecutive years (2016 to 2019) in January, when daily atmospheric temperatures rarely exceed 45°C. Sampling points were georeferenced using a Trimble handheld GPS (Juno SB series) equipped with the ESRI software ArcPad 10. For the collection of airborne dispersing microorganisms, we exposed for 5 days to the open air six 0.22-μm pore-size Nuclepore filters (4.7-cm diameter; Whatman, Maidstone, UK) fixed on initially sterile petri dishes that were deposited at five different sites on top of the Dallol dome (Fig. 1a to c). Additionally, we collected airborne particles (including microbes) by manually pumping ∼2 to 3 m^3^ air through another filter placed in a filtration system. The same filter was additionally exposed to the open air on the campsite at the southwest of the Dallol dome (Fig. 1a and d) for 11 days. Filters were kept and transported in closed petri dishes back to the laboratory and stored at −20°C until use.

Brine samples for molecular analysis of microbial diversity were sequentially filtered *in situ* through 30-μm and 0.22-μm-pore-diameter filters with due care to avoid external contamination; filters retaining the 0.22- to 30-μm-size-fraction biomass were fixed in ethanol and stored at −20°C as previously described (48, 51). Brines were also collected in sterile 50- to 100-mL glass bottles filled to the rim and sealed with rubber caps to prevent further oxidation. Physicochemical parameters were measured *in situ* with a YSI Professional Series Plus multiparameter probe for temperatures up to 70°C and with a Hanna HI93530 temperature probe (working range, −200 to 1,000°C) and a Hanna HI991001 pH probe (working range, −5 to 105°C; pH range, −2.00 to 16.00) at higher temperatures. Salinity was measured *in situ* with a refractometer on 1:10 dilutions in Milli-Q water and in the laboratory by weighting in triplicate the total solids after heat drying 1-mL aliquots at 120°C for at least 24 h.

Water activity and chaotropicity were measured experimentally in previous analyses (48, 51). Briefly, water activity was measured on native samples at room temperature (25°C) using an HC2-AW probe and HP23-AW-A indicator (Rotronic AG) calibrated at 23°C using the AwQuick acquisition mode (error per measure, 0.0027). Chaotropicity was determined according to the temperature of gelation of ultrapure agar (a proxy for biomolecules) mixed with a solution of Dallol brine using the spectrometric assay developed by Cray et al. (56). Experimental measures were congruent with calculated chaotropicity values estimated following the work of Cray and coworkers (79) based on the abundance of dominant Na, K, Mg, Ca and Fe cations in our samples and, given the local hydrochemistry, assuming that they essentially form chlorine salts (NaCl, KCl, MgCl_2_, CaCl_2_, and FeCl_2_).

### DNA purification and 16/18S rRNA gene amplicon sequencing.

DNA was purified from biomass retained on 0.2-μm filters using the Power Soil DNA isolation kit (MoBio, Carlsbad, CA, USA) with a UV-irradiated Erlab CaptairBio DNA/RNA PCR workstation. Samples were allowed to rehydrate overnight at 4°C in the kit resuspension buffer. DNA from the six aerosol-retaining filters exposed on the Dallol dome top were pooled on the same column of the last kit purification step to increase DNA yield. DNA was resuspended in 10 mM Tris-HCl, pH 8.0, and stored at −20°C. Bacterial and archaeal 16S rRNA gene fragments of approximatively 290 bp encompassing the V4 hypervariable region were PCR amplified using U515F (5′-GTGCCAGCMGCCGCGGTAA) and U806R (5′-GGACTACVSGGGTATCTAAT) primers. PCRs were conducted in 25 μL, using 1.5 mM MgCl_2,_ a 0.2 mM concentration of each deoxynucleoside triphosphate (dNTP) (PCR nucleotide mix; Promega), a 0.1 μM concentration of each primer, 1 μL of purified DNA, and 1 U of the hot-start *Taq* Platinum polymerase (Invitrogen, Carlsbad, CA, USA). Amplification reactions proceeded for 35 cycles (94°C for 15 s, 50 to 55°C for 30 s, and 72°C for 90 s), after a 2-min denaturation step at 94°C and before a final extension at 72°C for 10 min. Eukaryotic 18S rRNA gene fragments including the V4 hypervariable region were amplified using primers EK-565F (5′-GCAGTTAAAAAGCTCGTAGT) and 18S-EUK-1134-R-UNonMet (5′-TTTAAGTTTCAGCCTTGCG). Primers were tagged with different molecular identifiers (MIDs) to allow multiplexing and subsequent sequence sorting. All DNA samples processed for this study (Ctrl-Dome, Ctrl-Camp, 8Gt, and 8Ass) yielded amplicons in direct PCRs; all control reactions without template DNA were negative. Amplicons were visualized after gel electrophoresis and ultrasensitive GelRed nucleic acid gel stain (Biotium, Fremont, CA, USA) on a UV-light transilluminator. Amplicons from five independent PCRs for each sample were pooled and purified using the QIAquick PCR purification kit (Qiagen, Hilden, Germany). DNA concentrations were measured using the Qubit double-strand DNA (dsDNA) HS assay (Invitrogen). Equivalent amounts of amplicons were multiplexed and sequenced using paired-end (2 × 300 bp) MiSeq Illumina technology (Eurofins Genomics NGS Lab, Constance, Germany). Some eukaryotic amplicon samples (8Gt and 8Gt.MB3, 8Ass and 8Ass.MB3, 9Ass.Isl and 9Ass.Isl-CT) were sequenced as replicates.

### Sequence and phylogenetic analyses.

To properly compare sequence data of this and previous studies for which sequences were treated using an *ad hoc* pipeline (48), we reanalyzed raw sequences from all samples listed in Table S1 following the same pipeline using QIIME2 (80). Primers and MIDs were trimmed with cutadapt (81). Demultiplexing was performed using the “cutadapt demux-paired” function to look for MIDs at the beginning of sequences and properly attribute them to samples. Paired-end sequences were denoised, dereplicated, and chimera filtered using the DADA2 algorithm version 2018.2.6 (dada2 denoised-paired function) (82). Reads were trimmed to retain only high-quality sequences based on quality plots. Optimal trimming parameters for high resolution were determined using the complementary tool FIGARO (83). Amplicon sequence variants (ASVs), which we consider synonyms of operational taxonomic units (OTUs) defined at 100% sequence identity, were assigned to phylogenetic taxa using the SILVA reference database (release 138) (84) prior to reconstructing relative abundance histograms. Diversity (Simpson), richness (Chao1), and evenness indices were determined using the R package Vegan (85).

In addition, we placed in a reference phylogenetic tree the archaeal sequences identified in the Ctrl-Camp and Ctrl-Dome aerosols and the only OTU sequence identified on a Dallol chimney wall in another study (57). To do so, we added these sequences using MAFFT-linsi v7.38 (86) to previously generated reference alignments containing near-full-length archaeal 16S rRNA gene sequences from major reference taxa and previously obtained by Sanger sequencing from Lake Assale, the cave, and the salt plain, together with their closest BLAST hits (48). Poorly aligned regions were removed using TrimAl (87). A maximum-likelihood (ML) phylogenetic tree was constructed with IQ-TREE (88) using the GTR model of sequence evolution with a gamma law and taking into account invariable sites (GTR+G+I). Node support was estimated by ultrafast bootstrapping as implemented in IQ-TREE. Our shorter ASV/OTU sequences were then added to the reference alignment using the accurate -linsi “addfragments” option of MAFFT. This final alignment was split into two files (references and ASVs) before using the EPA-ng tool (https://github.com/Pbdas/epa-ng) to place ASVs in the reference trees reconstructed with IQ-TREE. The jplace files generated by EPA-ng were transformed into a Newick tree file with the Genesis library (https://github.com/lczech/genesis). Tree visualization was done with GraphLan (89).

### Cell and DNA degradation tests with hyperacidic brines.

We selected the brine collected from the Dallol pond 7DA9, pH −0.34 (Table S1; Fig. 4), to carry out the following experiments. First, we observed by epifluorescence microscopy (Zeiss Axioplan 2) cell-containing samples stained with the DNA-intercalating agent SYTO 9 (Thermo Fisher) before and immediately after contact with 7DA9 brine (addition of 2 volumes). We used cultures of the bacterium Escherichia coli and the haloarchaeon Halobacterium salinarum and natural samples (salt plain at the base of the dome, PS; salt canyons’ cave hypersaline reservoir, Gt, 8Gt, and 9Gt; Lake Assale or Karum, 8Ass and 9Ass) mixed or not with Dallol’s brine (1/3 of final volume). The pH of the mixed solution was always ≤0 as monitored by pH paper strips with a narrow range (0 to 6). Culture media and pure brine of Dallol were also observed as controls. Second, we measured the DNA concentrations of different culture amounts of E. coli and *H. salinarum* as well as a natural cell-containing PS brine sample before and after incubation with 7DA9 brine for 2, 5, and 30 min. Well-grown cell cultures (0.5 to 2 mL) and the PS sample (10 mL) were filtered onto 0.2-μm-pore-size filters (2.5-cm filter diameter) using small Millipore filtration systems, incubated with 500 μL of 7DA9 brine deposited on their surfaces, and, upon vacuum aspiration of the brine, rinsed with 2 mL of Milli-Q water. DNA was purified from filters using the PowerSoil DNA isolation kit (MoBio, Carlsbad, CA, USA). DNA concentrations were measured using Qubit dsDNA HS (Invitrogen).

Finally, we also attempted to PCR amplify 16S rRNA genes from cultures (E. coli and *H. salinarum*) and several natural PS, Gt, and Ass samples before and after contact with a solution mimicking Dallol brine chemistry (solution D: ATCC medium 112 without agar supplemented with 25% NaCl and acidified with HCl to a final pH of ≤0). Cell pellets were resuspended in solution D by vortexing and immediately centrifuged or neutralized prior to centrifugation by adding 2 volumes of either standard TE solution (1:1 mix of 10 mM Tris [pH 8] and 0.5 M EDTA [pH 9]) or TE+ solution (1:1 mix of 1 M Tris [pH 8] and 0.5 M EDTA [pH 9]), which raised the pH from ≤0 to 5 to 6 and to 7 to 8, respectively. After homogenization, the mixes were centrifuged 10 min at 10,000 × *g*, the supernatant was discarded, and the pellets were resuspended in 10 mM Tris, pH 8. After a freeze-thaw (at 42°C) cycle, the suspensions were microwaved (three times for 10 s each, mild conditions) to facilitate cell lysis. For PCR, we used archaeon-specific (21F, 5′-TTCCGGTTGATCCTGCCGGA; Ar109F, 5′-ACKGCTGCTCAGTAACACGT) and bacterium-specific (27F, 5′-AGAGTTTGATCCTGGCTCAG) forward primers with the prokaryotic reverse primer 1492R (5′-GGTTACCTTGTTACGACTT). Amplification reactions were performed for 35 cycles (94°C for 15 s, 50 to 55°C for 30 s, and 72°C for 90 s), after a 2-min denaturation step at 94°C and before a final extension at 72°C for 7 min. Amplicons were visualized after gel electrophoresis and staining with ultrasensitive GelRed nucleic acid gel (Biotium) on an UV light transilluminator.

### SEM and elemental mapping.

SEM analyses were carried out on liquid brine samples deposited onto 0.1-μm-pore-size filters (Whatman) by vacuum aspiration and briefly rinsed with 0.1-μm-filtered and autoclaved Milli-Q water. Filters were vacuum dried and stocked in a dry atmosphere until they were sputter coated with carbon for SEM observations. SEM analyses were performed using a Zeiss Ultra-55 field emission gun (FEG) SEM. Secondary electron (SE2) images were acquired at an accelerating voltage of 2.0 kV and a working distance of ∼3.0 mm. For chemical mapping, backscattered electron images were acquired using an angle-selective backscattered (AsB) detector at an accelerating voltage of 15 kV and a working distance of ∼3.0 mm. Elemental maps were generated from hyperspectral images (HyperMap) by energy dispersive X-ray spectrometry (EDXS) using an EDS QUANTAX detector. EDXS data were analyzed using the ESPRIT software package (Bruker).

### FISH.

FISH was carried out on brines (containing cells or not) subjected to identical protocols. To reproduce the FISH experiment of Gómez et al. to detect potential *Nanohaloarchaeota* in Dallol brines (57), we used the CY3-labeled probe Narc1214 (5′-CCGCGTGTATCCCAGAGC-3′) (90) using the hybridization conditions described previously (57). We included several hybridization and autofluorescence controls. In addition to the hyperacidic Dallol dome brines 7DA13 and DAL4, we included a sample from the salt plain (PS) unambiguously containing *Nanohaloarchaeota* (48) (Fig. 2) and a blank filter (negative control). Since *Nanohaloarchaeota* are usually epibionts of bigger cells (90–92), we used brines prefiltered through 20- or 50-μm nylon mesh to get rid of large mineral particles prior to retaining potential particles on 0.2-μm filters (25 to 50 mL). We then incubated the filters on 2 to 3 mL of 4% formaldehyde (methanol free, ultrapure; Polysciences, Inc., Warrington, PA, USA) for ∼1 h, rinsed them with 1× phosphate-buffered saline (PBS), applied vacuum for a few seconds to dry them, and stored them at 4°C in sterile petri dishes until use.

Immediately before hybridization, filters were dried again at 46°C before being cut into three pieces for hybridization with, respectively, no probe (negative control), the nonspecific probe CY3-NonEub (Non-Bact338, 5′-ACTCCTACGGGAGGCAGC-3′) (Thermo Fisher Scientific, Waltham, MA, USA) (59), and the *Nanohaloarchaeota*-specific probe CY3-Narc1214 (Eurofins Genomics, Germany). We added to filter sections 20 μL of hybridization buffer (180 μL of NaCl [5 M], 20 μL of Tris [1 M, pH 8.1], 300 μL of formamide, 1 μL of 10% SDS, 499 μL Milli-Q water) containing 50 ng of probe. Hybridization was done at 46°C for 90 min in a homogenized atmosphere. Filters were rinsed for 15 min in 50 mL of rinsing solution (1.02 mL NaCl [5 M], 1 M Tris-HCl [pH 8], 0.5 M EDTA [pH 8]; preheated at 48°C) and allowed to dry on absorbing paper. All solutions were 0.2-μm filtered and autoclaved; all dilutions were done using DNA-free water. Filter sections were then incubated with SYBR Gold for 15 min in the dark at room temperature, rinsed with Milli-Q water, and let dry. Hybridized filters were mounted between two glass slides with a drop of CitiFluor AF3 (Agar Scientific).

Hybridized samples were first observed under an Olympus BX51 epifluorescence microscope using the specific light filters U-MNIB3 (excitation, 470 to 495 nm; emission, 510 nm) and U-MWIG3 (excitation, 530 to 550; emission, 575 nm) and then under an Olympus FluoView FV1000 CLSM with a spectral resolution of 2 nm and a spatial resolution of 0.2 mm. Fluorescence images were taken with sequential excitation at wavelengths of 405, 488 and 543 nm by collecting the emitted fluorescence between 425 to 475, 500 to 530 and 560 to 660 nm, respectively. Additionally, we carried out hybridization of PS samples with other control probes (93): S-D-Arch-0915-a-A-20 (ARC915-CY3; 5′-GTGCTCCCCCGCCAATTCCT-3′), S-K-Eury-0498-a-A-14 (EURY498-FITC-; 5′-CTTGCCCRGCCCTT-3′) and the fluorescein-labeled NonEub (Non-Bact338, 5′-ACTCCTACGGGAGGCAGC-3′) combined with 2 different fluorophores, the cyanine CY3 and fluorescein isothiocyanate (FITC), as negative control. Samples were incubated at 46°C for 2 h with 5 ng/μL probe in 0.9 M NaCl, 20 mM Tris HCl (pH 8), 0.01% SDS containing 35% formamide. Samples were then washed for 15 min at 48°C in 20 mM Tris (pH 8.5), 8.5 mM EDTA, 0.01% SDS, 0.084 mM NaCl, soaked in cold water for a few seconds, and air dried. Samples were then incubated with the DNA-intercalating agent DAPI (4′,6-diamidino-2-phenylindole) (Sigma; 1 μg/mL) for 1 min, washed for a few seconds in cold water and left to dry. Hybridized cells, covered by CitiFluor AF3 (Agar Scientific), were examined under a Zeiss Axioplan 2 imaging epifluorescence microscope using specific filters for different wavelength emission and/or by confocal laser scanning microscopy (CLSM) using a FluoView FV1000 microscopy with a spectral resolution of 2 nm and a spatial resolution of 0.2 μm (Olympus, Tokyo, Japan).

### Data availability.

16S rRNA gene amplicon Illumina sequences have been deposited in the GenBank Short Read Archive with BioProject number PRJNA541281.
