# Supplementary material for: Active Microbial Airborne Dispersal and Biomorphs as Confounding Factors for Life Detection in the Cell-Degrading Brines of the Polyextreme Dallol Geothermal Field
Source: mBio. 2022 Apr 6;13(2):e00307-22. doi: 10.1128/mbio.00307-22 (PMC9040726; doi:10.1128/mbio.00307-22)
Supplement: TABLE S1 [file mbio.00307-22-st001.pdf]

Table S1. List of samples from the Dallol area featured in this study. RI, refractometer-inferred salinity, refers to the percentage (w/v) of salt measured in sity with a refractometer. ORP, oxido-reduction potential; Salinity was also estimated with total solids weight after water evaporation (dry weight measured in triplicates). DO, dissolved oxygen; ORP, oxidation-reduction potential; TS, total solids; SD, standard deviation; FISH, fluorescence in situ hybridization; epifOM, epifluorescence optical microsccopy; CSLM, confocal scanning laser microscopy; n. a., not applicable; n.d, not determined; metabar., metabarcoding (amplicon sequencing); abio, abiotic sample (molecular methods to reliably detect life failed).

Sample	Coordinates	Collection date	Physicochemical parameters								Analyses carried out in this study				Reference
			Temp (°C)	pH	DO (%)	ORP (mV)	RI salinity (%)	Salinity TS (g/l) ± SD	Water activity	Chaotro-picity	16S/18S rRNA metabar.	FISH - epifOM, CSLM	SEM	Cell/DNA degradation	
DALLOL AREA BIOAEROSOLS															
CTRL-CAMP	14.134230N 40.172676E	15.01.2019 to 25-01.2019	~25-53	n.a.	n.a.	n.a.	n.a.	n.a.	n.a.	n.a.	X				This study
CTRL-DOME (6 filters)	(1) 14.14741N 40.18708E	15.01.2019 to 19.01.2019	~25-53	n.a.	n.a.	n.a.	n.a.	n.a.	n.a.	n.a.	X				This study
	(2) 14.14741N 40.18933E		~25-53	n.a.	n.a.	n.a.	n.a.	n.a.	n.a.	n.a.					This study
	(3) 14.141845N 40.175090E		~45-65	n.a.	n.a.	n.a.	n.a.	n.a.	n.a.	n.a.					This study
	(4) 14.142000N 40.175140E		~25-53	n.a.	n.a.	n.a.	n.a.	n.a.	n.a.	n.a.					This study
	(5) 14.142900N 40.174849E		~25-53	n.a.	n.a.	n.a.	n.a.	n.a.	n.a.	n.a.					This study
	(6) 14.142900N 40.174849E		~25-53	n.a.	n.a.	n.a.	n.a.	n.a.	n.a.	n.a.					This study
Lake Assale or Karum															
Ass	14.089567N 40.348583E	23.01.2016	26.2	6.7	53.0	97.7	20.0	360 ± 11	0.771	n.d	X*				Belilla et al. 2019
8Ass	14.089567N 40.348583E	15.01.2018	22.0	6.5	14.0	221.0	31.0	363 ± 11	0.718	n.d	X			X	This study
9Ass	14° 3'13.13"N 40°22'51.97"E	23.01.2019	30.6	6.7	46.3	184.3	29.0	302 ± 4	0.718	n.d	X*			X	Belilla et al. 2021
Salt canyon's cave brine															
Gt	14.229704N 40.289952E	18.01.2016	n.d	n.d	n.d	n.d	30	n.d	0.728	n.d	X*				Belilla et al. 2019
7Gt	14.229704N 40.289952E	11.01.2017	29.0	6.3	9.5	117.2	30.0	323 ± 19	0.729	-18.3	X*				Belilla et al. 2019
8Gt	14.229704N 40.289952E	16.01.2018	22.0	6.5	15.2	262.1	30.0	330 ± 10	0.731	-57.3	X			X	This study
9Gt	14.229704N 40.289952E	16.01.2019	25.5	5.9	46.0	240.3	30.0	325 ± 5	0.725	n.d	X*			X	Belilla et al. 2021
Salt plain at the dome base															
PS**	14.2250194N 40.2889000E	19.01.2016	n.d	n.d	n.d	n.d	n.d	n.d	n.d	n.d	X*	X	X	X	Belilla et al. 2019
PS3	14.22912N 40.297935E	19.01.2016	29.3	4.21	n.d	n.d	20.0	n.d	n.d	n.d	X*				Belilla et al. 2019
Round Mountain - Western Canyon Lakes															
WCL1	14.241511N 40.268053E	18.01.2019	29.3	4.85	32	165.4	30	404 ± 20	0.719	-27.1	X*				Belilla et al. 2019
WCL3	14.242139N 40.267853E	18.01.2019	30.5	4.93	30	130.0	n.d.	412 ± 18	0.690	-17.3	X*				Belilla et al. 2019
WCL5	14.242111N 40.267164E	18.01.2019	29.3	5.1	26	119.8	32	337 ± 11	0.725	-15.6	X*				Belilla et al. 2019
Dallol hydrothermal ponds															
7DA9	14.241528N 40.29884E	08.01.2017	31.9	-0.34	5.5	369	43	379 ± 17	0.708	-8.2	abio			X	Belilla et al. 2019
7DA13	14.235495N 40.298135E	11.01.2017	50.2	-0.43	7.5	324	35.0	342 ± 18	0.723	-24.8	abio	X	X		Belilla et al. 2019
DAL4***	14.23916N 40.297059E	17.01.2016	30.1	-0.78	1.3	393	n.d.	405 ± 19	n.d.	n.d.	abio	X	X		Belilla et al. 2019

* 16S rRNA amplicon sequence data produced in previous studies and re-analyzed for standardized comparison in this work

** Original wet salt pan fragment subsequently maintained in laboratory mesocosm at salt-saturating conditions.

*** Brine with salt fragments from the wall delimiting pond DAL4D (Belilla et al, 2019).
