# Supplementary material for: Active Microbial Airborne Dispersal and Biomorphs as Confounding Factors for Life Detection in the Cell-Degrading Brines of the Polyextreme Dallol Geothermal Field
Source: mBio. 2022 Apr 6;13(2):e00307-22. doi: 10.1128/mbio.00307-22 (PMC9040726; doi:10.1128/mbio.00307-22)
Supplement: FIG S1 [file mbio.00307-22-sf001.pdf]

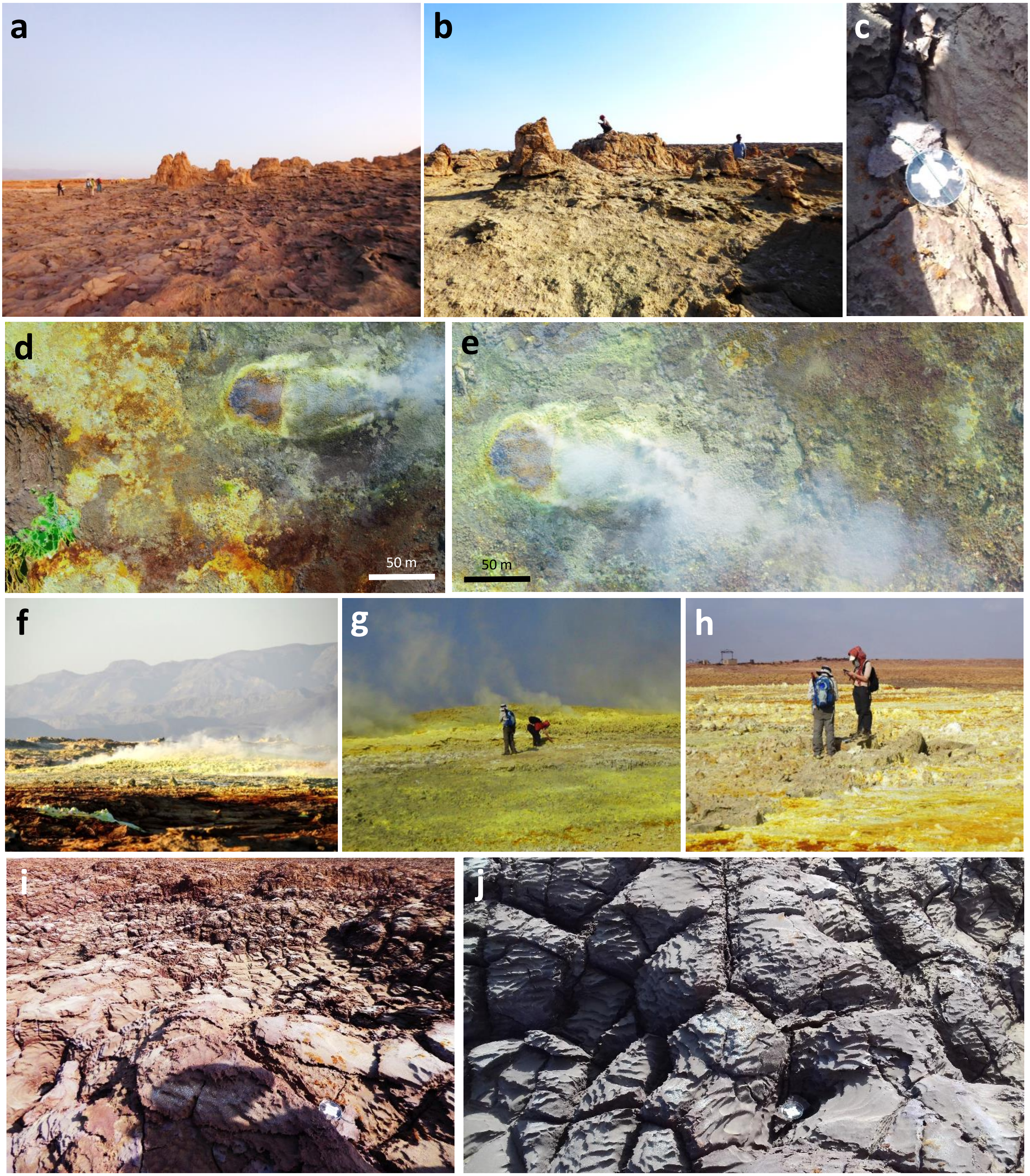


FIG S1 Bioaerosol collecting areas on the geothermal Dallol dome and dust accumulation in the Chocolate formation during one year.

**a**) Overview of the ancient hydrothermal chimney area close to the tourist path chosen to expose filters 1 and 2. **b**) Filters 1 and 2 were exposed on top on two extinct chimneys, hidden from tourist sight. **c**) Filter 2 in place. **d-e**) Drone image of the hottest active degasing area (45-60°C air temperature) chosen to place filter 3. **f**) Lateral view of the active degasing area. **g**) Placing filter 3 for exposure. **h**) Placing filter 4, for exposure ~30m N-E of filter 3, between salt boulders. **i**) Filter 5 exposed on the 'Chocolate' formation on top of the dome. **j**) Filter 6, exposed a few meters away from filter 5 in the same Chocolate formation.

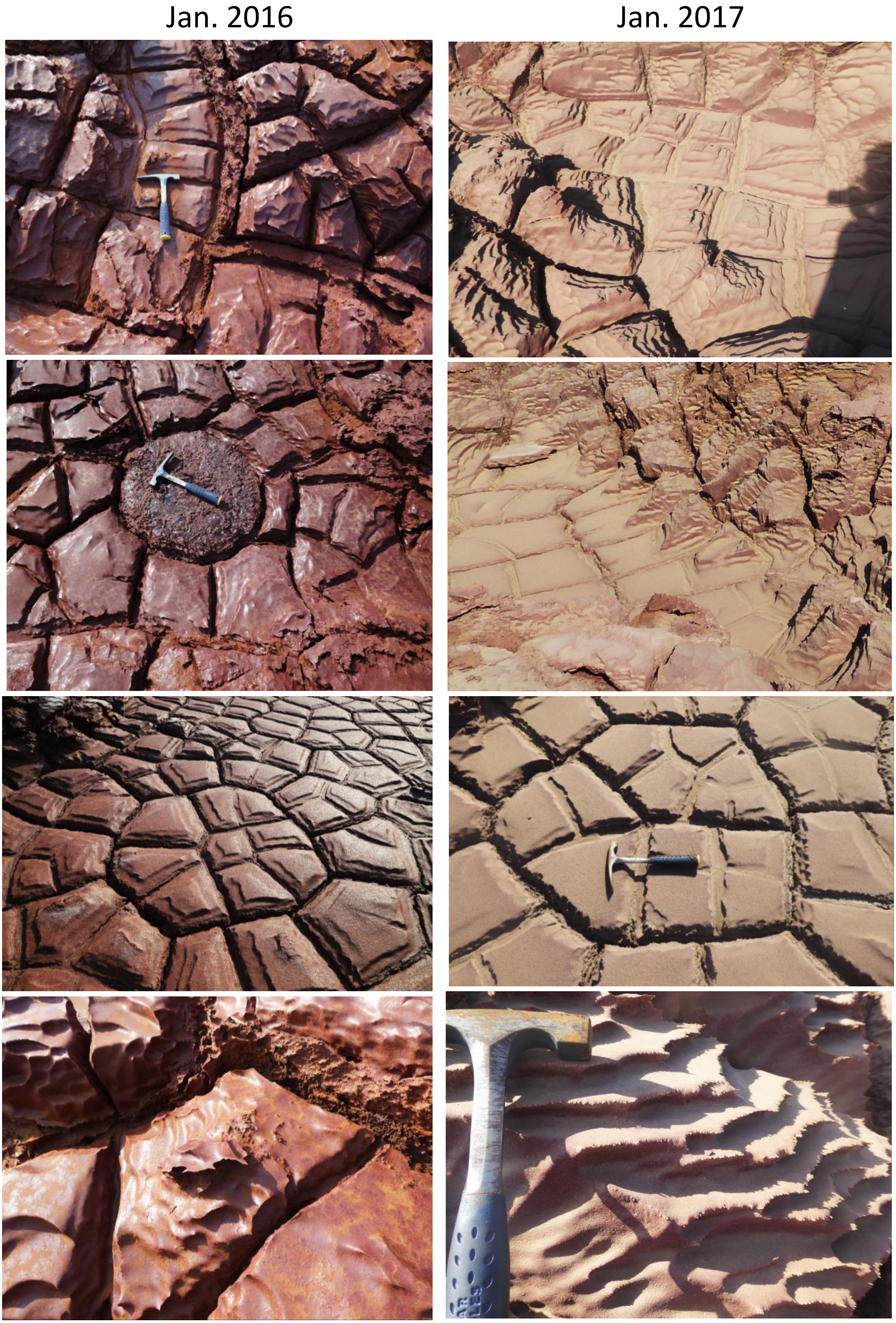


FIG S1 (cont.) Bioaerosol collecting areas on the geothermal Dallol dome and dust accumulation in the Chocolate formation during one year.
k) Photographs of the ‘Chocolate’ formation taken in January 2016, after a rain episode (left) and in January 2017. Note the whitish-yellowish layer of dust deposited by the wind.
