# Supplementary material for: Active Microbial Airborne Dispersal and Biomorphs as Confounding Factors for Life Detection in the Cell-Degrading Brines of the Polyextreme Dallol Geothermal Field
Source: mBio. 2022 Apr 6;13(2):e00307-22. doi: 10.1128/mbio.00307-22 (PMC9040726; doi:10.1128/mbio.00307-22)
Supplement: FIG S2 [file mbio.00307-22-sf002.pdf]

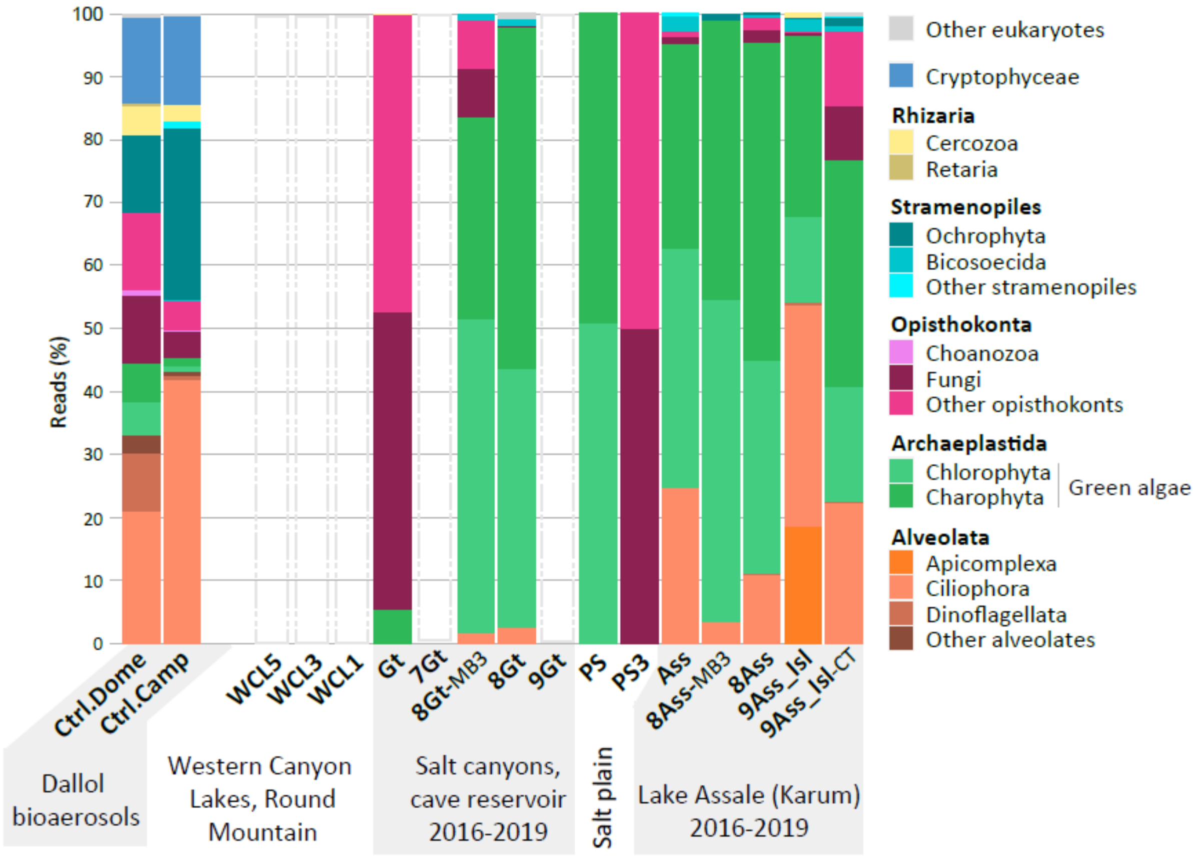

FIG S2 Presence, diversity and relative abundance of eukaryotic 18S rRNA gene amplicon sequences detected in Dallol bioaerosols compared with life-hosting environments in the Dallol surroundings. Empty boxes correspond to samples for which amplicon sequences could not be obtained. Assignation and frequency of the different ASVs/OTUs are given in Table S4. 8Gt and 8Gt-MB3, 8Ass and 8Ass-MB3 and 9Ass-Isl and 9Ass-Isl-CT are replicates sequenced in independent Illumina runs. Note that eukaryotes are only detected sporadically in Gt samples from the cave reservoir depending on the year and the fluctuating level (and associated conditions) of the brine. Note also that in Gt and PS3 samples, the number of ASVs was extremely small (5 and 2, respectively, see Table S2), notably making the opisthokont sequences in these two samples suspect of exogenous contamination (these ASVs were also found in aerosols).
