# Supplementary material for: Active Microbial Airborne Dispersal and Biomorphs as Confounding Factors for Life Detection in the Cell-Degrading Brines of the Polyextreme Dallol Geothermal Field
Source: mBio. 2022 Apr 6;13(2):e00307-22. doi: 10.1128/mbio.00307-22 (PMC9040726; doi:10.1128/mbio.00307-22)
Supplement: FIG S3 [file mbio.00307-22-sf003.pdf]

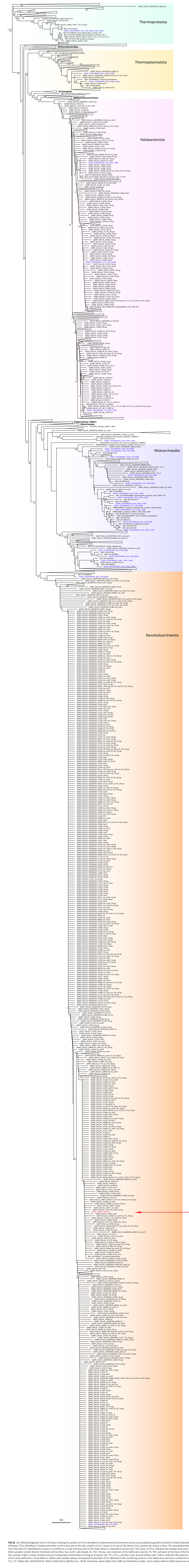

FIG S3 16S rRNA phylogenetic tree of Archaea showing the position of OTUs identified in polyextreme environments at and surrounding the geothermal dome of Dallol (Danakil Depression, Ethiopia). OTUs identified in bioaerosols either at the camp site at the Salt canyons (Ctrol_Camp) or on top of the dome (Ctrol_dome) are shown in blue. The nanohaloarchaeal sequence from the only OTU identified by Gomez et al (2019) on a small chimney wall on the Dallol dome is indicated in red (arrow). The name of OTUs indicates the samples they were identified in. Most samples contain diverse microbial communities: Ass, AssPJ, Lake Assale; Gt, 7Gt, 7Gt-pp, cave reservoir at the Dallol salt canyons; PS, PS3, salt plain at the base of the Dallol dome. A few samples might contain incipient aerosol-transported communities forming an incipient soil: 7YLs,7YL-s, soil-like crusts around Yellow Lake; 7DA5s, sediment-like deposit at the dome. OTUs rarely detected in a few Dallol or Yellow Lake samples always correspond to abundant OTUs detected in life-containing systems in the Dallol area and were condisered contaminants: YL2, 7YL Yellow lake; DAL6A7DA10, 7DA14, Dallol dome (Belilla et al., 2019). Bootstrap values higher than 50% are indicated at nodes. Some clades without Dallol sequences were collapsed.
