# Supplementary material for: Active Microbial Airborne Dispersal and Biomorphs as Confounding Factors for Life Detection in the Cell-Degrading Brines of the Polyextreme Dallol Geothermal Field
Source: mBio. 2022 Apr 6;13(2):e00307-22. doi: 10.1128/mbio.00307-22 (PMC9040726; doi:10.1128/mbio.00307-22)
Supplement: FIG S4 [file mbio.00307-22-sf004.pdf]

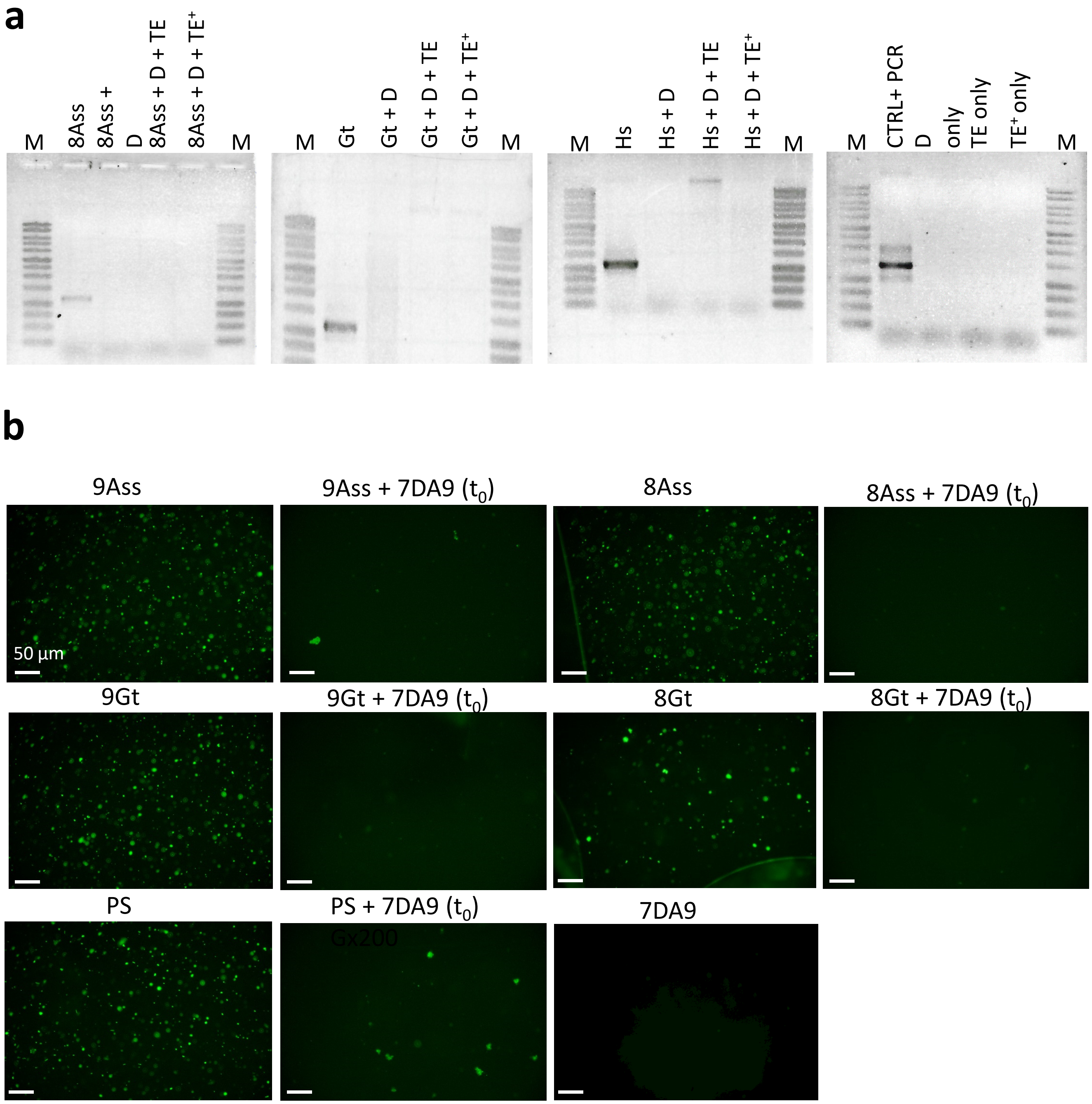

FIG S4 Additional examples of cell and DNA-degrading effects of hyperacidic Dallol brines.

a) 16S rRNA gene amplifying test on resuspended cell pellets from natural samples and cultures after contact with acidified (pH~0) hypersaline medium (D) mimicking Dallol brines. From left to right, panels correspond to treatment of cells from the Lake Assale (8Ass), the cave reservoir at the salt canyons (Gt), ***Halobacterium salinarum,*** and positive and negative controls of the experiment. Contact with D lasted around 11 min (vortex followed by 10 min centrifugation) in samples + D, but less than 30 s in samples neutralized with TE or TE+ solutions (see text). Pellets were resuspended in 10 mM Tris pH 8.5 prior to PCR amplification. M, size markers. **b**) SYTO9-induced epifluorescence of various brines at the Dallol dome and surrounding systems before and immediately after (t0) adding 2 volumes of 7DA9 brine. 9Ass, Lake Assale (2019); 8Ass, Lake Assale (2018); 9Gt, cave reservoir (2019); 8Gt, cave reservoir (2018); PS, salt plain; 7DA9, Dallol pond hyperacidic brine (2017). Scale bar, 50 µm.
