# Supplementary material for: Active Microbial Airborne Dispersal and Biomorphs as Confounding Factors for Life Detection in the Cell-Degrading Brines of the Polyextreme Dallol Geothermal Field
Source: mBio. 2022 Apr 6;13(2):e00307-22. doi: 10.1128/mbio.00307-22 (PMC9040726; doi:10.1128/mbio.00307-22)
Supplement: FIG S5 [file mbio.00307-22-sf005.pdf]

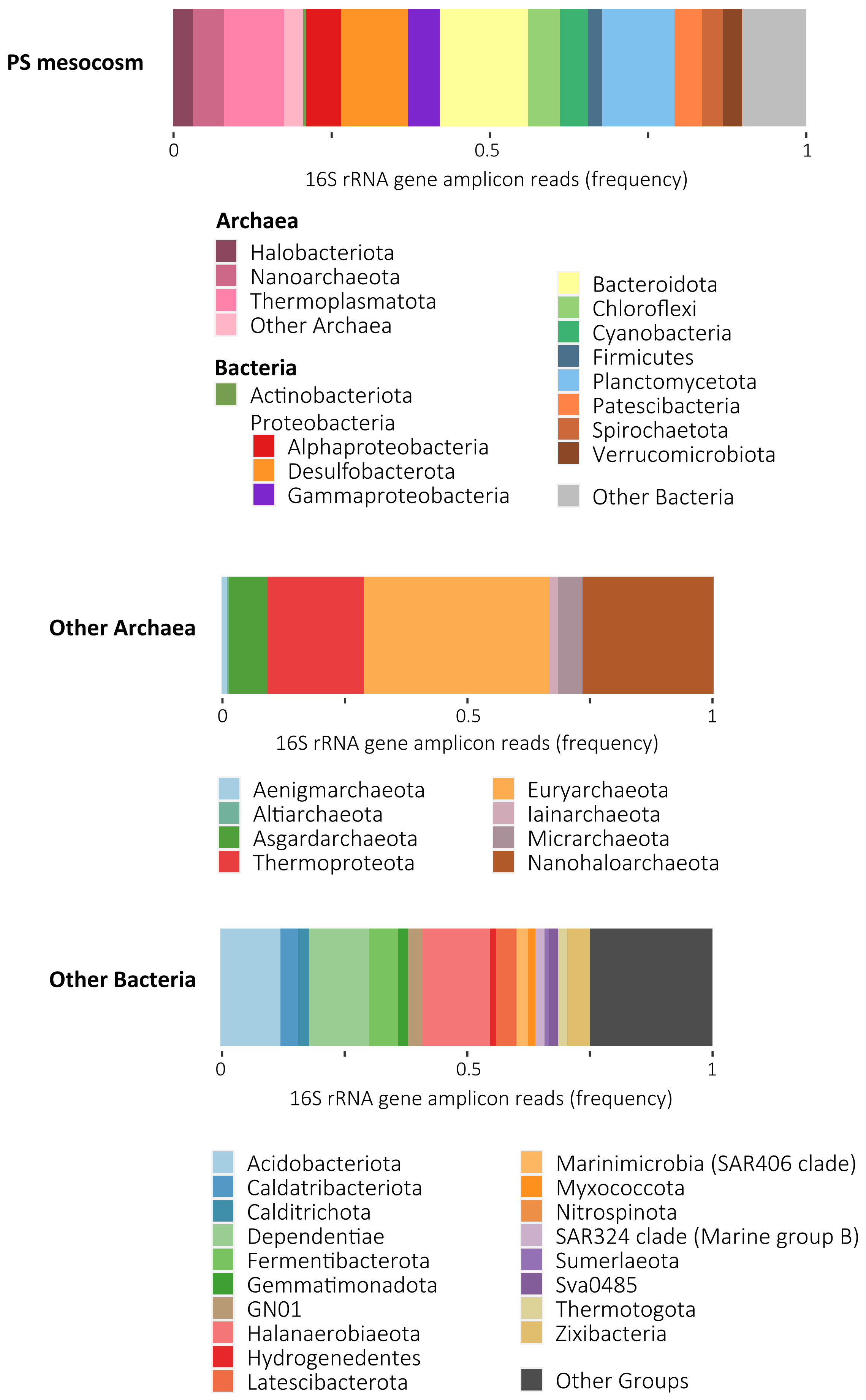

FIG S5 16S rRNA gene based prokaryotic diversity present in the PS sample maintained in a laboratory mesocosm at the time when FISH experiments were carried out. The upper panel correspond to dominant taxa, the panels below provide details about the diversity of less abundant taxe. See also Table S2D for additional details.
