# Supplementary material for: Active Microbial Airborne Dispersal and Biomorphs as Confounding Factors for Life Detection in the Cell-Degrading Brines of the Polyextreme Dallol Geothermal Field
Source: mBio. 2022 Apr 6;13(2):e00307-22. doi: 10.1128/mbio.00307-22 (PMC9040726; doi:10.1128/mbio.00307-22)
Supplement: FIG S6 [file mbio.00307-22-sf006.pdf]

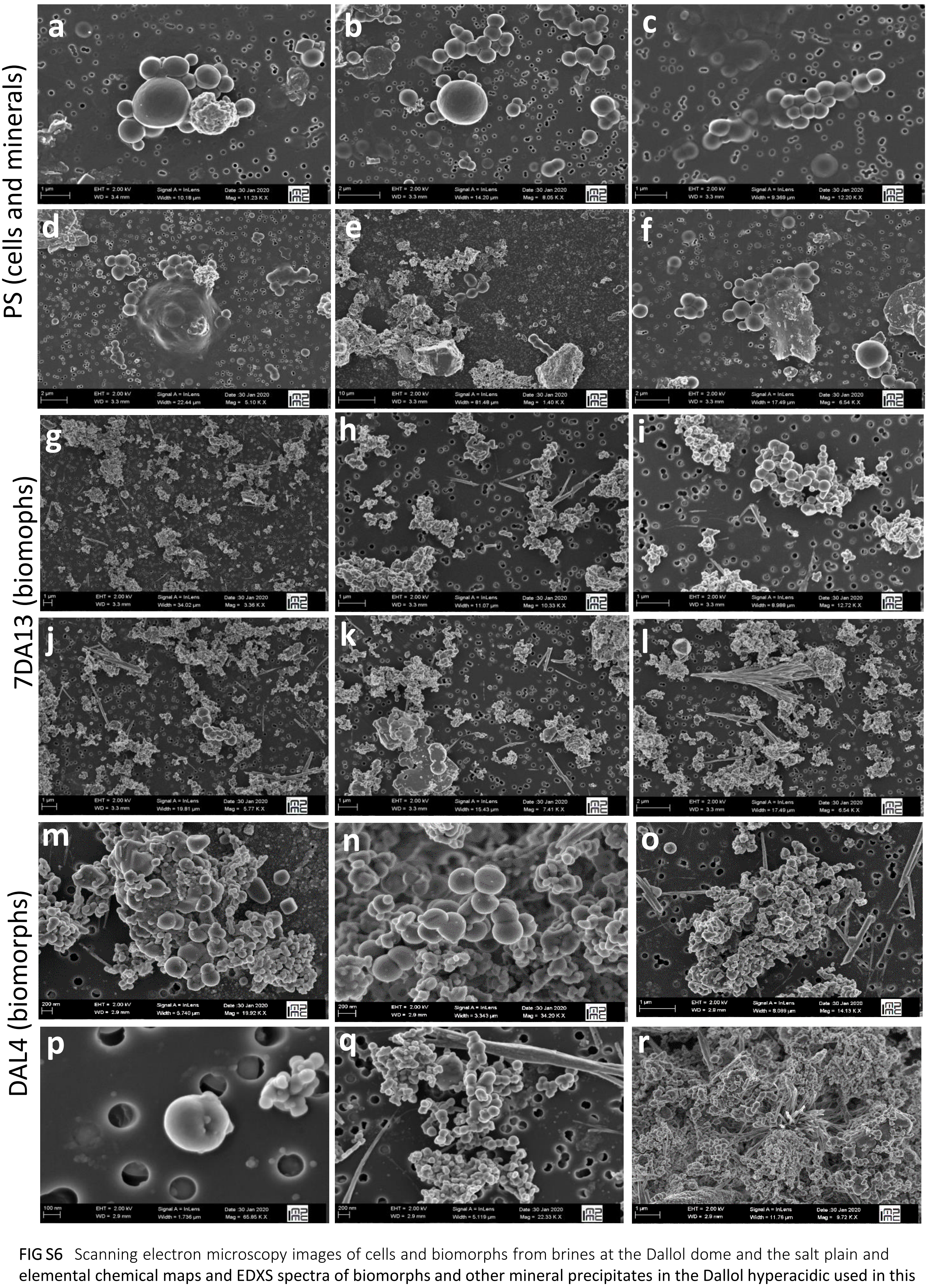

FIG S6 Scanning electron microscopy images of cells and biomorphs from brines at the Dallol dome and the salt plain and **elemental chemical maps and EDXS spectra of biomorphs and other mineral precipitates in the Dallol hyperacidic used in this study**. Samples from the salt plain (PS; panels a-f) and from the Dallol dome hyperacidic brines 7DA13 (g-l) and DAL4 (m-r).

s

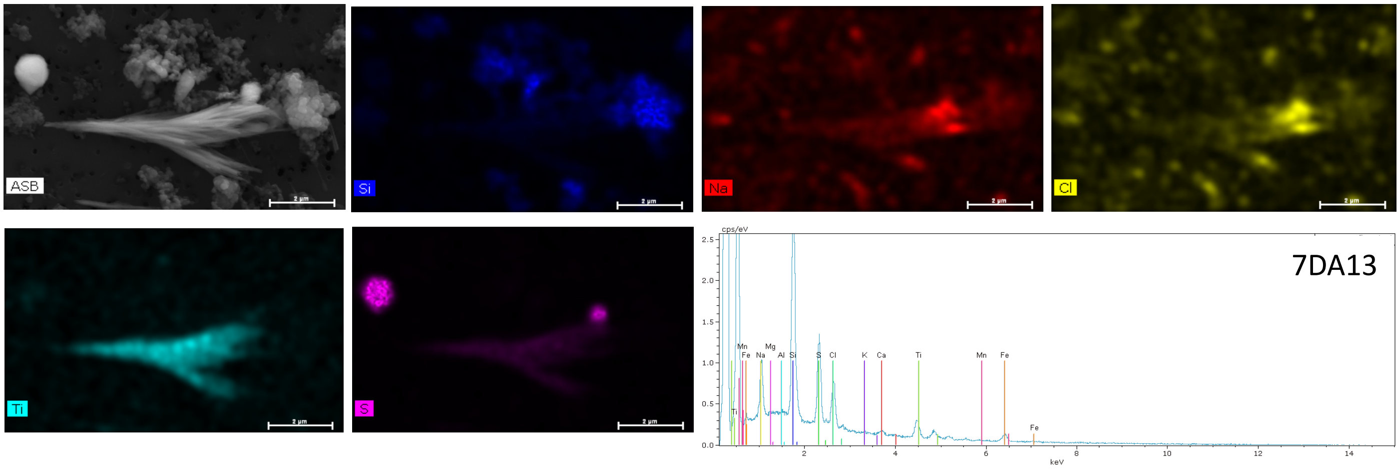

t

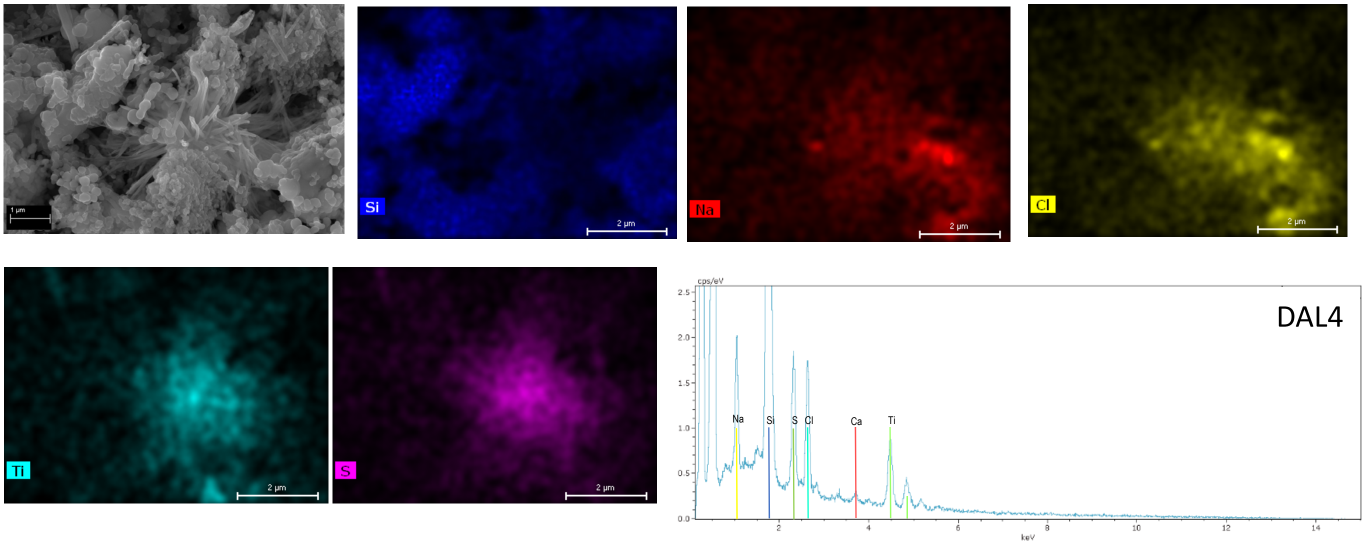

u

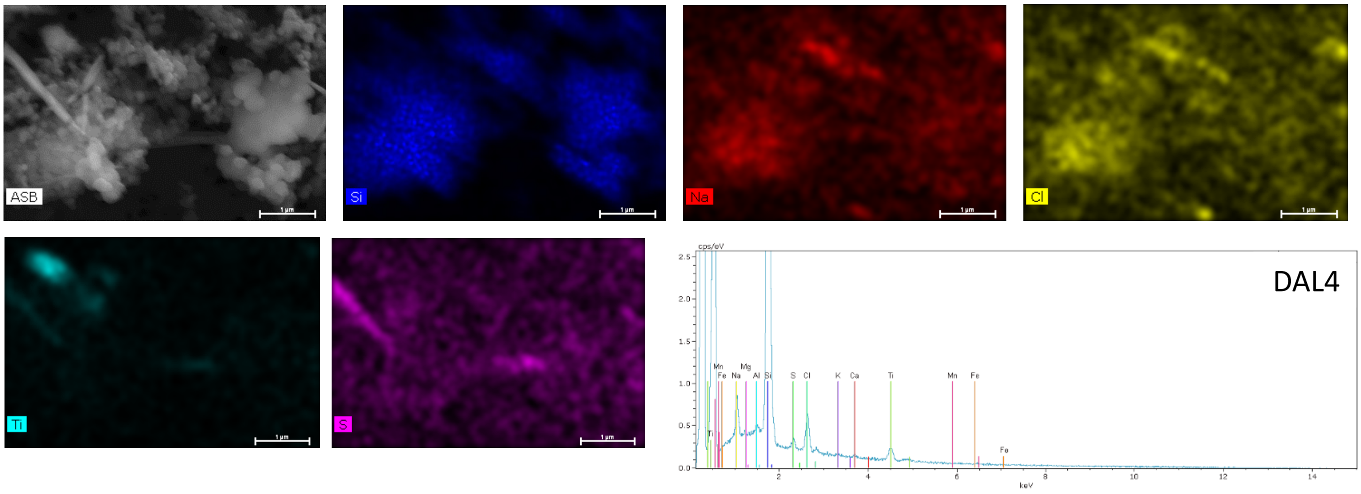

FIG S6 (cont.) Scanning electron microscopy images of cells and biomorphs from brines at the Dallol dome and the salt plain and **elemental chemical maps and EDXS spectra of biomorphs and other mineral precipitates in the Dallol hyperacidic used in this study**. Samples with chemical maps correspond to 7DA13 (s) and DAL4 (t-u). SEM photographs were taken using In Lens (t) or AsB detectors.
