# Supplementary material for: Active Microbial Airborne Dispersal and Biomorphs as Confounding Factors for Life Detection in the Cell-Degrading Brines of the Polyextreme Dallol Geothermal Field
Source: mBio. 2022 Apr 6;13(2):e00307-22. doi: 10.1128/mbio.00307-22 (PMC9040726; doi:10.1128/mbio.00307-22)
Supplement: FIG S7 [file mbio.00307-22-sf007.pdf]

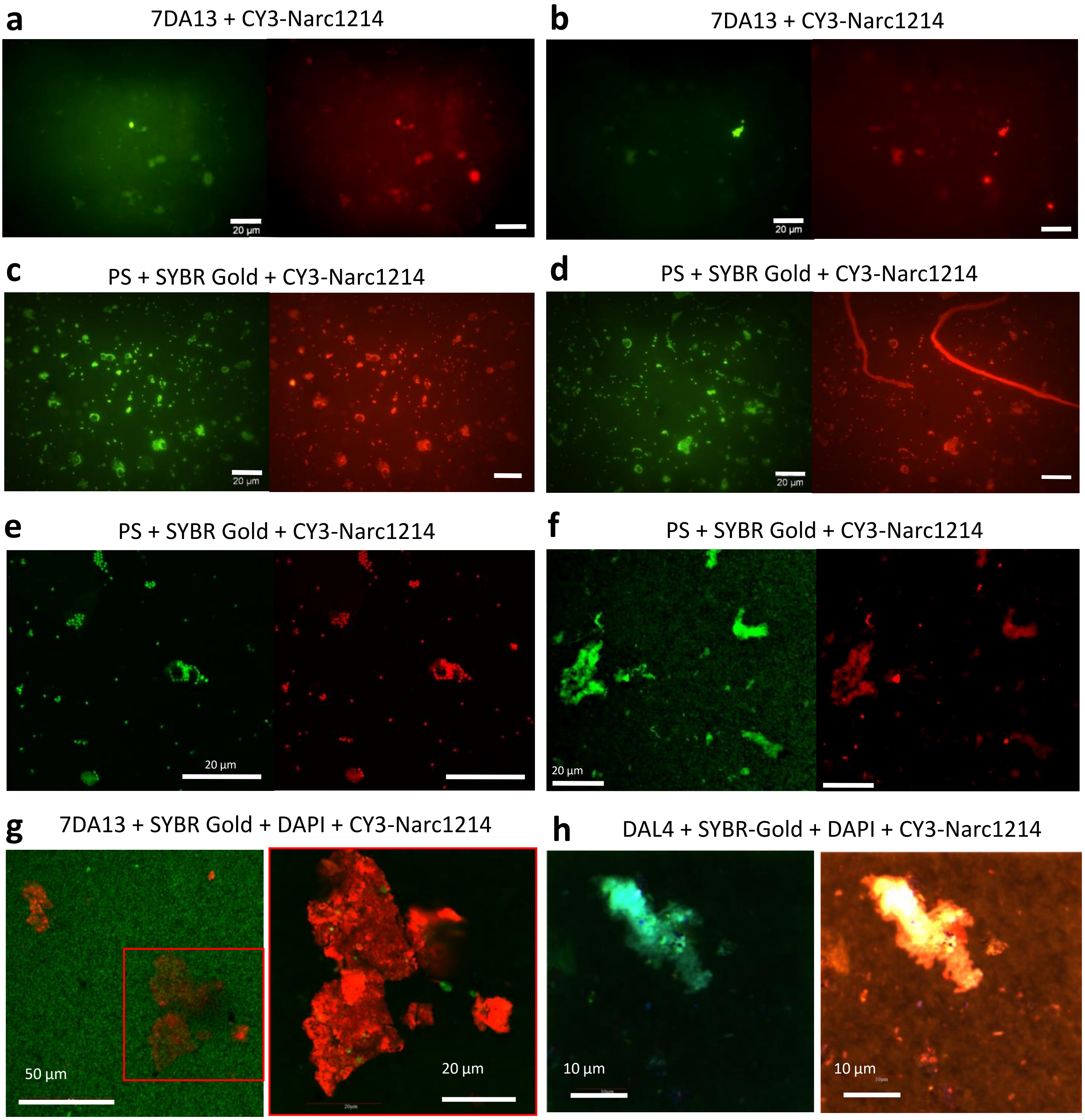

FIG S7 Additional images of FISH experiments on Dallol hyperacidic brines and the salt plain ecosystem using the Nanohaloarchaeota-specific probe CY3-Narc1214. In all cases, the DNA intercalating agent SYBR Gold was used for fluorescent staining (green, left panels). The red fluorescence of CY3-Narc1214 is shown on the right panels. The name of the sample and the probe used are indicated. **a-b**) epifluorescence microscopy of FISH experiments on Dallol brine 7DA13. **c-f**) epifluorescence microscopy of FISH experiments on salt plain (PS) sample. **g**) CLSM images of FISH hybridization experiments on 7DA13 brine. The left panel shows the superposition of SYBR Gold, DAPI and CY3-Narc1214 fluorescence (note the high background due to unspecific staining of the salt embedded filter). Right panel, magnification of the area framed in red and CY3-Narc1214 binding to mineral particles after increasing contrast. **h**) CLSM images of a FISH hybridization experiment on DAL4 brine. Left and right panels correspond to a superposition of the SYBR Gold, DAPI and CY3 fluorescence but the left panel was obtained in sequential mode. FISH hybridization conditions were the same as described by Gomez et al (2019).
