# Supplementary material for: Active Microbial Airborne Dispersal and Biomorphs as Confounding Factors for Life Detection in the Cell-Degrading Brines of the Polyextreme Dallol Geothermal Field
Source: mBio. 2022 Apr 6;13(2):e00307-22. doi: 10.1128/mbio.00307-22 (PMC9040726; doi:10.1128/mbio.00307-22)
Supplement: FIG S8 [file mbio.00307-22-sf008.pdf]

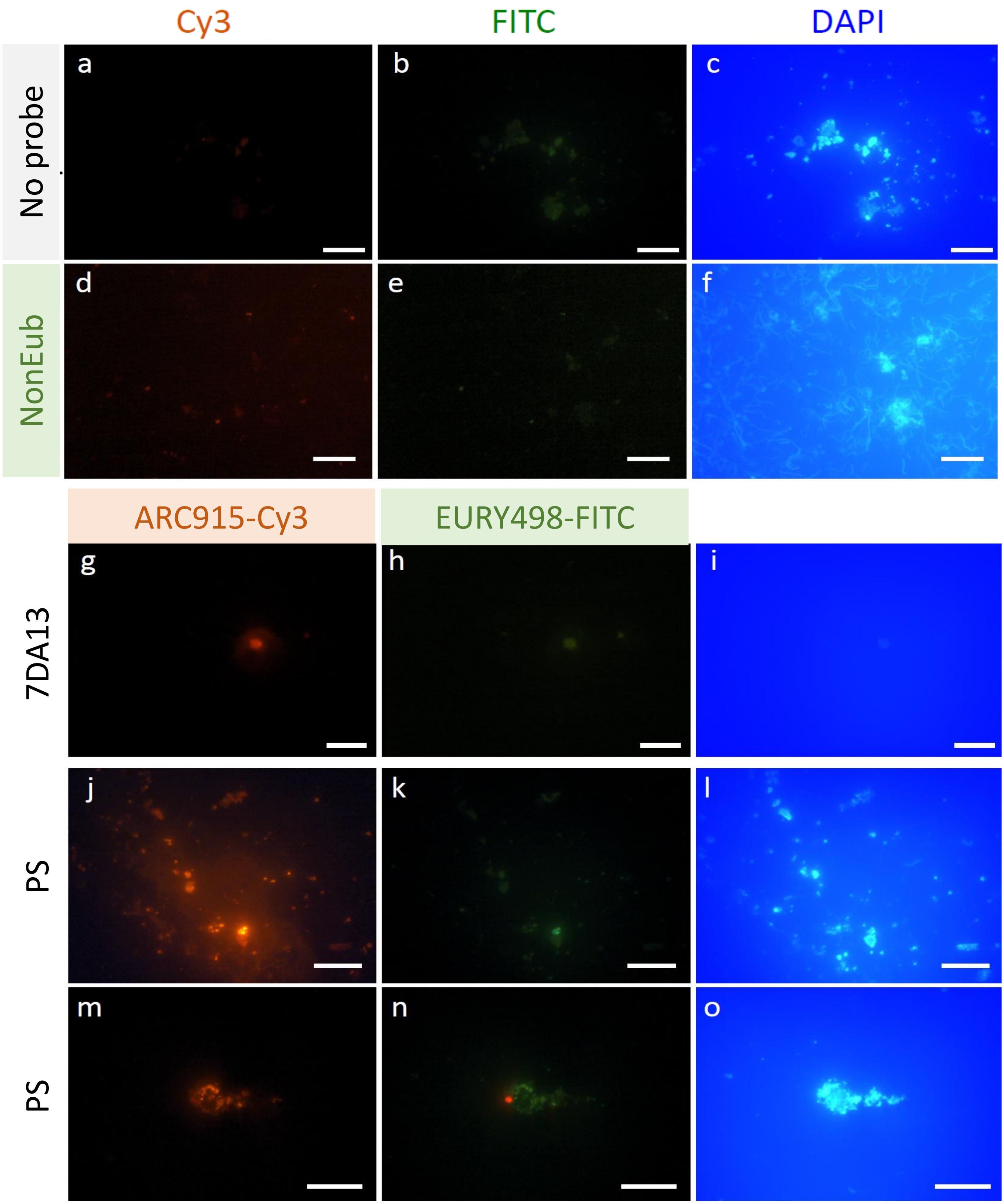

FIG S8 Comparative epifluorescence microscopy of salt plain (PS) mesocosm samples and Dallol 7DA13 hyperacidic brines in FISH experiments using control and archaea-specific probes. Hybridized samples were observed by epifluorescence microscopy (Zeiss Axioplan) using filters for the detection of Cy3 (left panels), FITC (central panels) and DAPI (right panels). Probes labelled with FITC and Cy3 are indicated in green and orange, respectively. DAPI staining is indicated in blue. For proper comparison, the light intensity at which all micrographs were taken was identical. a-f) Control FISH experiments on PS microbial cells hybridized with no probes (a-c) or with control probe nonBAC388 (complementary to a universal bacterial probe; d-f). Note the slight natural autofluorescence of cells observed with the FITC filter (b,e) and some autofluorescence of NonEub-hybridized cells with the Cy3 filter (d). g-o) Samples hybridized with the archaeal probes ARC915 and EURY498. g-i) Hybridization of 7DA13 brine. Most fields were fully dark but occasionally, mineral particles adsorbed the probes (DAPI staining low to none). j-o) Hybridization of archaeal PS cells with ARC915 and EURY498. Note also the red autofluorescence of a green algal Cell (n). Scale bars, 20 µm.
